# Microfluidic production of amiodarone loaded nanoparticles and application in drug repositioning in ovarian cancer

**DOI:** 10.1038/s41598-024-55801-3

**Published:** 2024-03-15

**Authors:** Asia Saorin, Gloria Saorin, Fahriye Duzagac, Pietro Parisse, Ni Cao, Giuseppe Corona, Enrico Cavarzerani, Flavio Rizzolio

**Affiliations:** 1 Department of Molecular Sciences and Nanosystems, Ca’Foscari University of Venice, Venezia-Mestre, Italy; 2 Department of Clinical Cancer Prevention, The University of Texas MD Anderson Cancer Center, Houston, TX USA; 3 Elettra-Sincrotrone Trieste S.C.p.A., Area Science Park, Strada Statale 14 km 163.5, Basovizza, 34149 Trieste, Italy; 4 CNR-IOM – Istituto Officina dei Materiali, Area Science Park, s.s. 14 Km 163.5, Basovizza, 34149 Trieste, Italy; 5 Immunopathology and Cancer Biomarkers Unit, Centro di Riferimento Oncologico di Aviano (CRO), IRCCS, Aviano, Italy; 6 Pathology Unit, Centro di Riferimento Oncologico di Aviano (C.R.O.) IRCCS, 33081 Aviano, Italy

**Keywords:** Ovarian cancer, CPT1A, Amiodarone, Microfluidics, Lipidic nanoparticles, Cancer, Nanoscience and technology

## Abstract

Amiodarone repositioning in cancer treatment is promising, however toxicity limits seem to arise, constraining its exploitability. Notably, amiodarone has been investigated for the treatment of ovarian cancer, a tumour known for metastasizing within the peritoneal cavity. This is associated with an increase of fatty acid oxidation, which strongly depends on CPT1A, a transport protein which has been found overexpressed in ovarian cancer. Amiodarone is an inhibitor of CPT1A but its role still has to be explored. Therefore, in the present study, amiodarone was tested on ovarian cancer cell lines with a focus on lipid alteration, confirming its activity. Moreover, considering that drug delivery systems could lower drug side effects, microfluidics was employed for the development of drug delivery systems of amiodarone obtaining simultaneously liposomes with a high payload and amiodarone particles. Prior to amiodarone loading, microfluidics production was optimized in term of temperature and flow rate ratio. Moreover, stability over time of particles was evaluated. In vitro tests confirmed the efficacy of the drug delivery systems.

## Introduction

Ovarian cancer is the third common gynaecological cancer after cervical and uterine, but it shows the highest mortality rate, and it represents the fifth cause of cancer death in women^1–3^. The term ovarian cancer includes more than 30 different types of cancer with epithelial ovarian cancer being the most common (90% of total)^4^ and showing a poorer prognosis^5,6^. Epithelial ovarian cancer can be further subdivided, with the subtype high grade serous ovarian cancer being the most diagnosed (70%) generally discovered at advanced stage and showing a low five-years survival rate (30%)^6,7^. Indeed, the disease remains quite asymptomatic until it metastasizes. Epithelial ovarian cancer dissemination is dependent on tumour cells acquiring the ability to resist anoikis and survive long enough to attach to metastatic sites within the peritoneal cavity or survive within ascites. Anoikis resistance drives a metabolic reprogram in towards a predilection for fatty acid oxidation^8,9^, obtained also by upregulation of one component of carnitine palmitoyltransferase system (CPT). CPT is a multiprotein complex responsible for the transport of long chain fatty acids across the inner and outer mitochondrial membrane and it consists of three components: CPT1, CPT2, and carnitine acylcarnitine translocase (CACT). CPT2 and CACT are located in the inner membrane of the mitochondria while CPT1 is located in the outer. CPT1 is the rate limiting step of fatty acid oxidation since it mediates the transformation of acyl-CoAs into acylcarnitines allowing fatty acid transport thought the outer mitochondrial membrane^10^. CPT2 is ubiquitously expressed in the body while CPT1 presents three isoforms: CPT1A (liver form), CPT1B (muscle form) and the recently discovered CPT1C (brain form)^11^. These isoforms of CPT1 are involved in different diseases such as cardiovascular diseases, metabolic syndrome, diabetes mellitus type 2, neuropsychiatric and neurological diseases and cancer^11,12^. In particular, CPT1A has found to be overexpressed in most ovarian cancer cell lines and primary ovarian serous carcinomas, to correlate with poor overall survival of ovarian cancer patients, and to be upregulated in cells cultured in suspension namely mimicking in vitro anoikis resistance^9,13^. Ovarian cancer cell lines were proven to be dependent on CPT1A mediated fatty acid oxidation for cell cycle progression since the inactivation of CPT1A decreased ATP levels and induced cell cycle arrest at G0/G1, moreover CPT1A deficiency also suppressed anchorage-independent growth^13^.

Therefore, targeting fatty acid oxidation upregulation could be a possible improvement in epithelial ovarian cancer therapy^14^. In the present study, we want to consider amiodarone, which is an antiarrhythmic agent, for its role as inhibitor of CPT1A. Amiodarone, approved by Food and Drug Administration (FDA) in 1985, still represents the most effective antiarrhythmic drug which acts blocking several ions channels such as sodium, potassium and calcium. It is used for the treatment of most prevalent type of atrial fibrillation and arrhythmia, but also in emergency settings, such as in case of acute myocardial infarction^15,16^. Beside this well-known use of amiodarone in cardiac patients, pre-clinical studies showed its actions as anti-cancer drug both as a single drug or in combination with chemotherapeutics reducing drug resistance in different kind of tumours^17–20^ included epithelial ovarian cancer^21^, but its mechanism of action as fatty acid oxidation inhibitor has not been considered. However, the limit in the repurposing of amiodarone is represented by its unfavourable toxicity profile derived by its lipophilicity, which makes it to accumulate in skin, lung, liver up to 100 days after the treatment^20^. Indeed, amiodarone treatment for long time (1 year or more) even with low doses can elicit adverse effects on thyroid, skin, eyes, and to the neurologic system^22^. Moreover, amiodarone doses in cancer treatment are far from being settled but a previous study evaluated its administration associated with doxorubicin or vinblastine in refractory breast cancer patients, determining significant side effects at the selected concentration^23^. Therefore, considering the importance of drug repurposing (only 5% of new cancer drugs reach the approval)^24^ and the fact that the other proposed CPT1A inhibitors demonstrated even higher toxicity^25,26^, it is of particular interest the development of delivery systems (DDSs) which could reduce side effects of the drug reducing off-target distribution. Moreover, DDSs are also useful for poor soluble drugs, like amiodarone , since solubility influences drug absorption and distribution and hence it is a key parameter related to the therapeutic efficacy. Among the different drug delivery systems, liposomes have proven their efficacy since 1995 with the approval of Doxil^®^, a PEGylated liposomal formulation of doxorubicin^27^. Several methods are available for the preparation of liposomal formulations, ranging from laboratory scale to Good Manufacturing Practice production for clinical batches. Microfluidics has been recently employed to prepare liposomes. Different microfluidic approaches are reported in literature among which micromixers have been recently developed. Micromixers can be employed to achieve liposomes with sizes within the optimal range for cancer therapies, making them suitable for exploiting the enhanced permeability and retentioneffect (diameter around 100 nm). A stream of alcoholic solution, containing the lipids, is intersected, and flanked by aqueous streams and then mixed in chips leading to liposome formation by self-assembly driven by the antisolvent activity of the aqueous stream. In micromixer, mixing is facilitated by micro-architectures on the surface of chip channels, which improve flow mixing, resulting in reduced dilution and higher throughput compared to previously developed microfluidic approaches^28,29^. Thanks to the precise control enabled by microfluidic technology, it is possible to monitor the mixing of fluid flows, thus allowing for the regulation of vesicle lamellarity, average size, and size distribution. Therefore, using microfluidics, homogenization steps that are required after other liposome production methods, are not needed. Reducing the steps required for liposomes production means decreasing the possible contamination of the samples, the loss of materials and the release of the drug from the liposomes.

In this study, the production of lipidic drug delivery systems through microfluidics was explored along with the evaluation of amiodarone effects on epithelial ovarian cancer cell lines both as free drug and encapsulated into drug delivery systems. Liposomal production through microfluidics was studied considering the effects of production parameters over final characteristics of liposomes that were studied both as empty and encapsulating amiodarone. The Doxil® formulation of liposomes was chosen for this work since it is already clinically approved^30^. The addition of amiodarone into lipids formulation determine the formation of amiodarone particles (AP) along with liposomes (AL), which were fully studied. Furthermore, the effects of amiodarone on the lipid metabolism of epithelial ovarian cancer cells were evaluated to better explore its potential as an inhibitor of CPT1A, confirming its activity. Amiodarone efficacy was assessed in ovarian cancer cells (A2780, Kuramochi and OVCAR-5), which being epithelial cell lines grow in adhesion, but it was also tested in anokis resistant cells, i.e. cells forced to grown in suspension. Indeed, anoikis resistant cells are considered to overexpress CPT1A and increase lipid metabolism. Moreover, amiodarone particles were also tested on SKOV-3 spheroids, which beyond being a model of anokis resistant cells, are also used to evaluate permeability of formulations.

To the best of our knowledge the present study is the first which focuses on the activity of amiodarone in ovarian cancer considering its activity as inhibitor of CPT1A and evaluating possible drug delivery systems. Moreover, amiodarone loaded liposomes were obtained for the first time with FDA approved  lipid formulation of Doxil^®^ and both liposomes and amiodarone particles showed higher drug content compared to previously published studies^31–33^.

## Results

### Microfluidic production of Doxil^®^ formulated liposomes

The production of Doxil formulated liposomes (DFL) (i.e. without amiodarone, also referred as “empty”) by the microfluidic setup was studied considering the influence over samples of different flow rate ratio (FRR) and temperature. The effects of temperature and FRR were studied since the first one was not properly addressed in literature while the latter is the microfluidic parameter that most affects liposome characteristics^28,29^. Lipid composition and aqueous media were chosen in view of in vitro experiments and clinically approved liposomal formulation.

#### Temperature effect over DFL

All the tested temperature conditions (see Materials and methods) allowed to obtain liposomes as proved by transmission electron microscopy (TEM) images reported in Fig. 2S.

**Figure 1:**
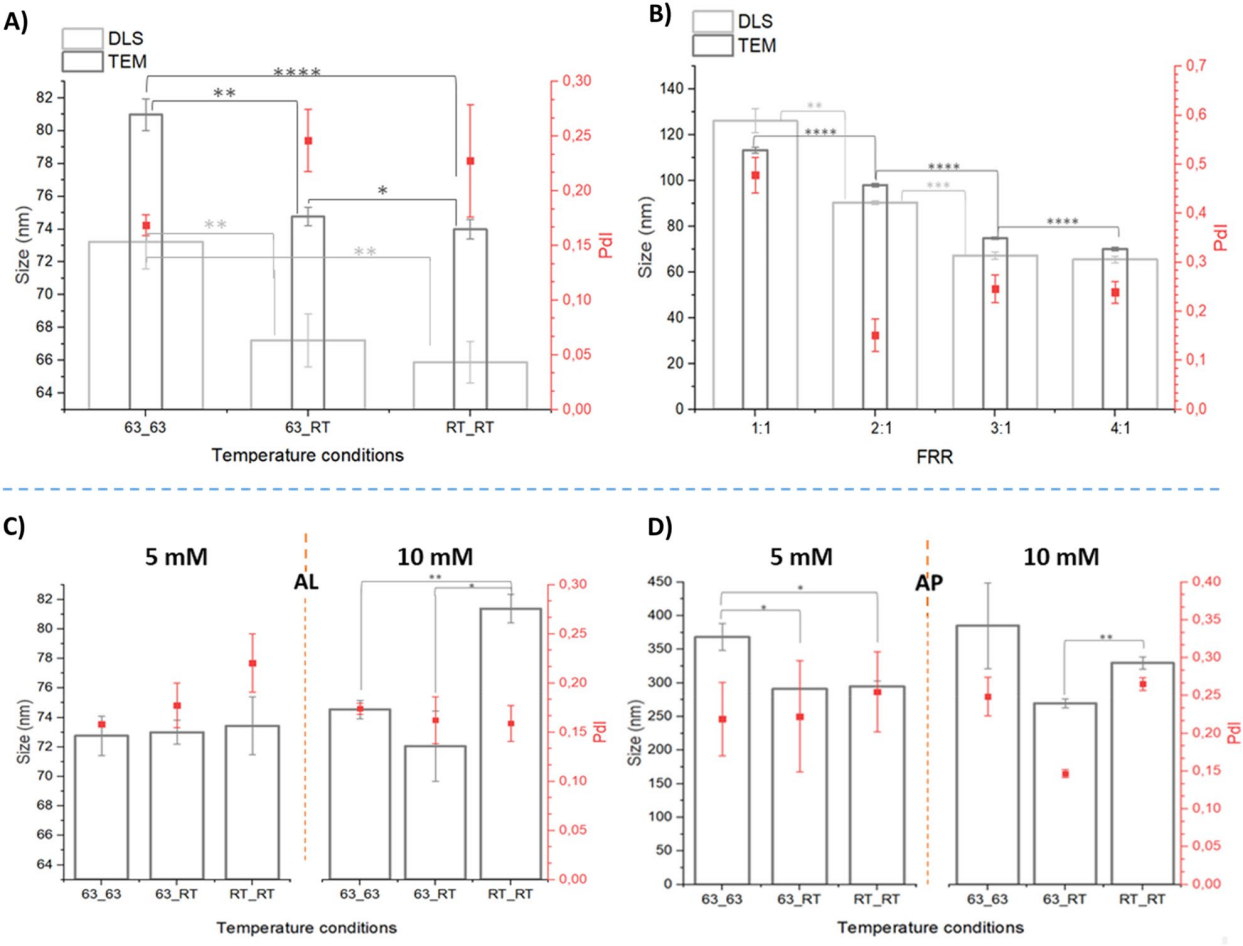
DLS and TEM data of DFL and ALP. Above: Average dimensions determined by DLS (light grey, standard deviation (SD) reported as error) and TEM (dark grey, standar error of the mean (SEM) reported as error) and polydispersity index (PdI) values, for DFL produced at (**A**) different temperature conditions, FFR 3:1, TFR = 1 ml/min, lipids concentration 10 mM and (**B**) different FRR, TFR = 1 ml/min, lipids concentration 10 mM, 63_RT. DLS and TEM showed same trends even though with higher sensitivity in case TEM technique. Below: DLS data; Z average and PdI of AL (**C**) and AP (**D**) samples at the three different temperature conditions 63_RT, 63_RT and RT_RT and with amiodarone concentration equal to 10 and 5 mM (FRR 3:1, TFR = 1 ml/min, lipids 10mM). (t-test, p-value * < 0.05, ** < 0.01,*** < 0.001, **** < 0.0001, when not reported no significative difference were found).

**Figure 2:**
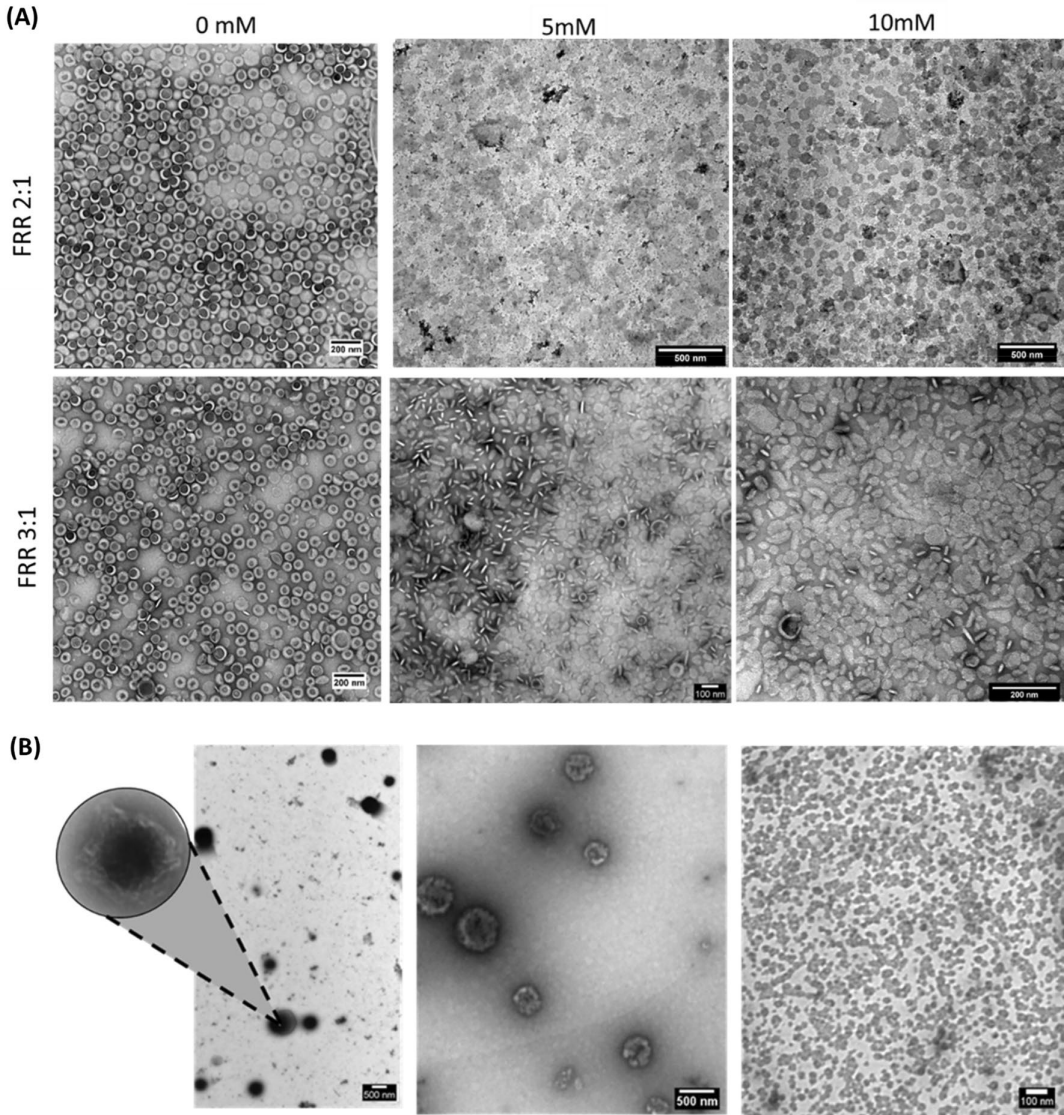
TEM images of ALP. (**A**) AL obtained at different amiodarone concentration and FRR (**B**) AP, on the left aggregates, on the right single NPs.

Temperature seems to influence the average diameters and distributions; indeed, its increment increases the average size, while the PdI value is below the quality threshold of 0.2 in the case of both reservoirs heated (Fig. 1). Proton nuclear magnetic resonance spectroscopy (H-NMR) proved that the nominal formulation of Doxil®, i.e. L-α-phosphatidylcholine (HEPC), cholesterol (CHO), 1,2-distearoyl-sn-glycero-3-phosphoethanolamine-N-[methoxy(polyethylene glycol)-2000] (DSPE-PEG) HEPC:CHO:DSPE-PEG 55:40:5, was maintained for all the temperature conditions (Fig. 2S). The yields of the lipids forming the DFL are always quite high with few values over 100% for 63_63 (heating both reservoirs) probably due to a concentration effect in the heated alcoholic reservoir (Fig. 2S). The condition 63_RT (heating only the alcoholic solution reservoir) is the most reproducible one in terms of lipid yields, comparable with method reproducibility (Fig. 2S, Table 2S). Indeed, it allows to avoid flow instability observed for RT_RT (both reservoirs unheated) and evaporation of the solvent determined for 63_63, therefore it has been selected for further studies.

#### FRR effect over DFL

**Figure 3:**
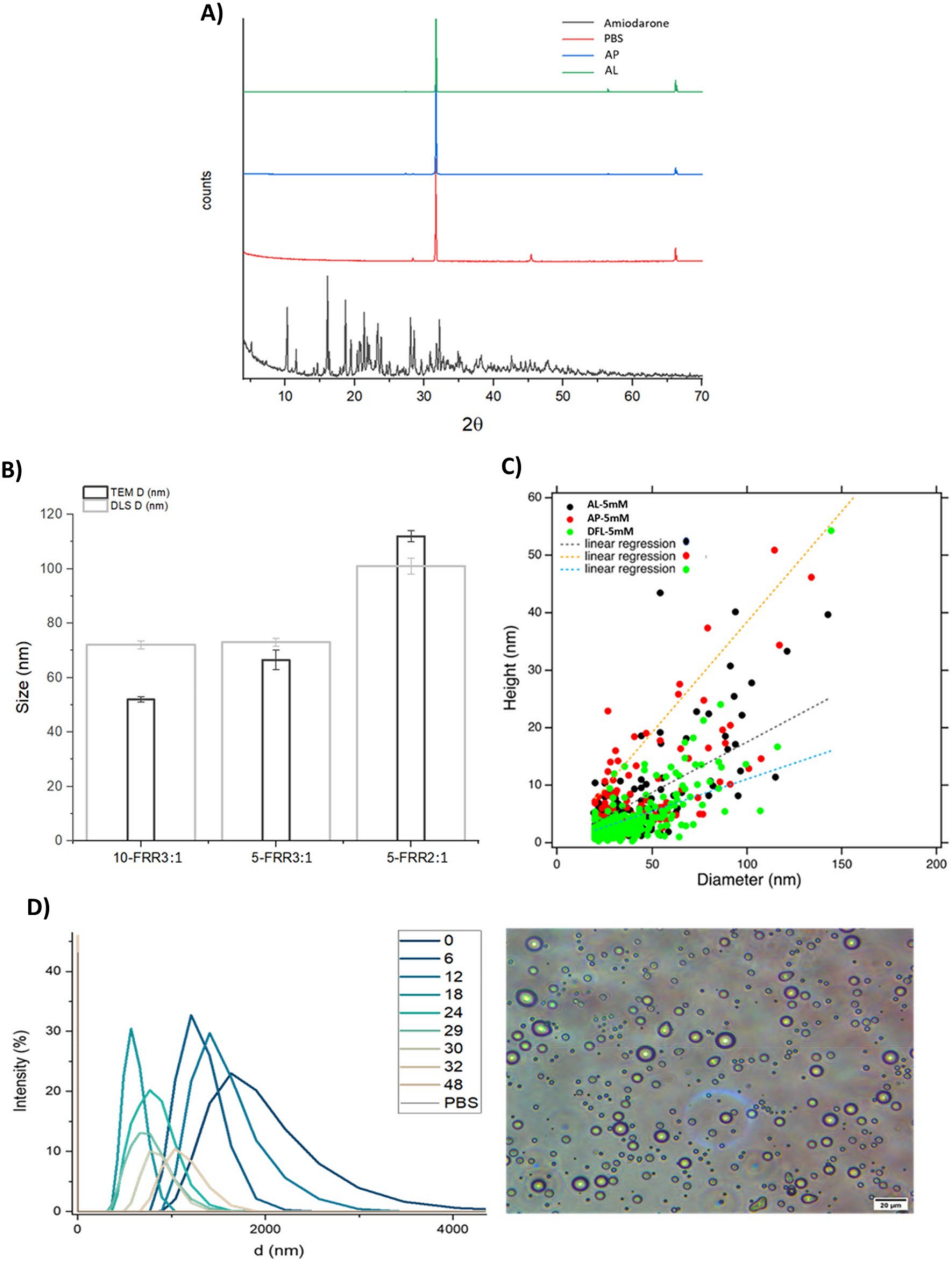
ALP characterization data. (**A**) XRD spectra AL (amiodarone liposomes) (green), AP (amiodarone particles) (blue), AM (amiodarone) drug powder (black) and PBS (red). (**B**) Size comparison between TEM and DLS data for AL samples obtained with FRR equal to3:1 and 2:1 63_RT, and amiodarone concentration of 10 and 5mM. (**C**) AFM analysis: hight-diameter correlation for Doxil formualated liposomes (DFL) (green), AL (black) and AP (red). (**D**) Characteristics of microfluidic 5mM AM FRR3:1 sample obtained in absence of lipids. On the left time course DLS analysis, on the right optical microscope image of fresh sample.

**Figure 4:**
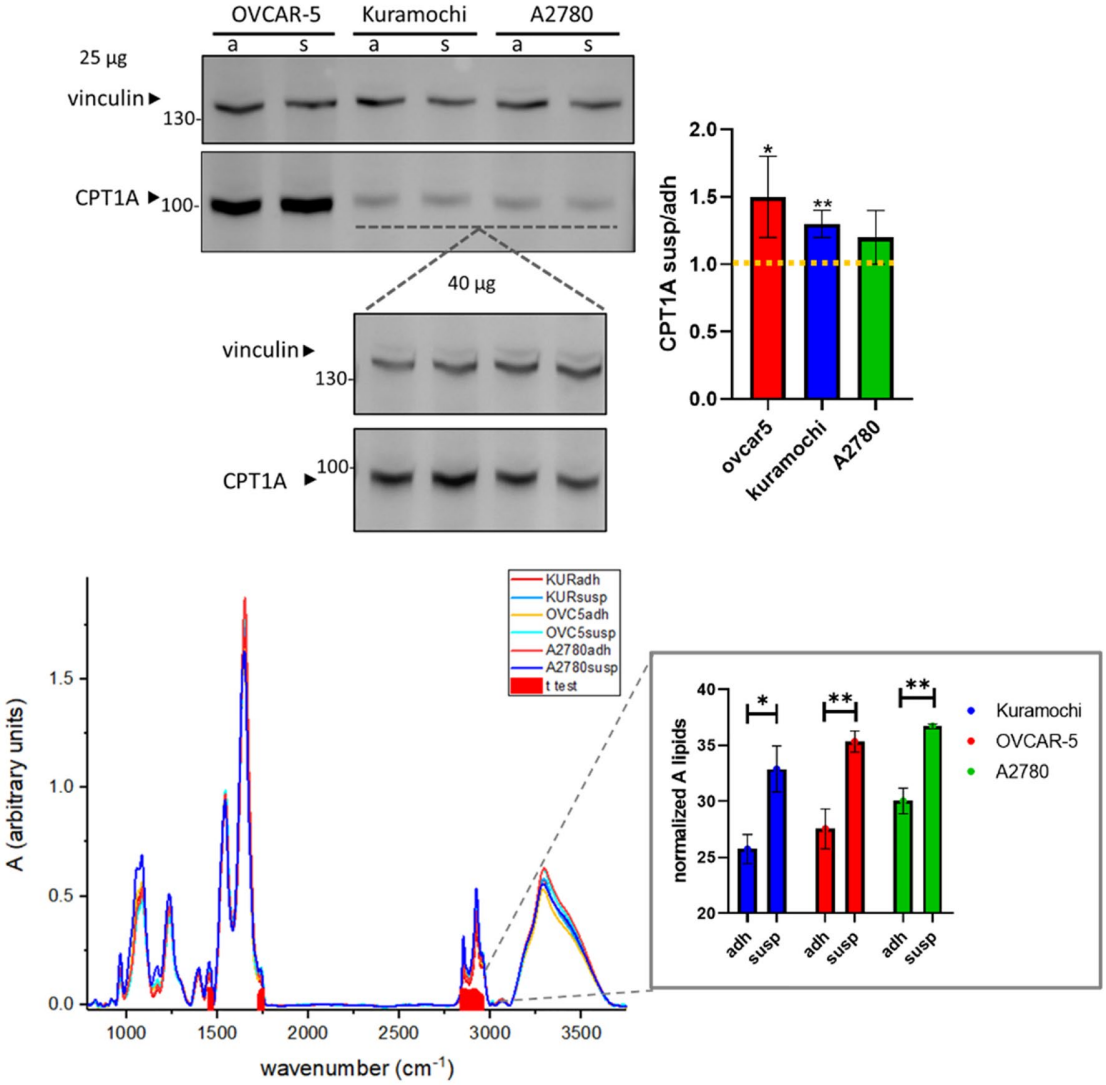
CPT1A expression and metabolic FTIR profile data. On the top, western blot of CPT1A (88kDa) and vinculin (used for normalization, 124kDa) corresponding to adhesion (a) and suspension (s) cultures of A2780, OVCAR-5 and Kuramochi obtained with the loading of 25µg of total proteins. Magnification of bands of A2780 and Kuramochi obtained with the loading of 40µg of total proteins. Original blots are presented in Supplementary Fig. 11S. Bar plots of the ratio of suspension to adhesion band intensities of CPT1A (t test, * p < 0.1, **p < 0.05). On the bottom, FT-IR spectra of cells highlighting significantly different spectral areas determined by t-test reported as false discovery rate (FDR) transformed by − log10 so that the more significant features are plotted higher on the graph. In the box integrated lipids areas (t test, * p < 0.05, **p < 0.01).

FRR has been varied (4:1, 3:1, 2:1, 1:1 expressed as DPBS:Ethanol solutions flow rate), maintaining the other parameters fixed (total flow rate(TFR) = 1 ml/min, 63_RT, lipids 10 mM, Doxil formulation). Both dynamic light scattering (DLS) analysis and TEM images assess the possibility to obtain liposomes with good characteristics using FRR 4:1, FRR 3:1 and FRR 2:1,with the best polydispersity index (PdI) in the latter FRR condition, revealing a trend of decreasing size associated with FRR increment (Fig. 1, Fig. 3S). Instead, FRR1:1 determined the formation of bimodal populations of vesicles, namely small unilamellar vesicles (SUV) and large unilamellar vesicles LUV (Fig. 3S). At low values of FRR, the presence of the micron sized peak is attributed to the presence of higher content of ethanol^28^, however lower FRR correspond even to an higher lipid content. Therefore, a formulation with higher content of lipids (20 mM) was used at FRR2:1 proving the formation of two populations of liposomes (Fig. 4S).

#### Stability over time of DFL

**Figure 5:**
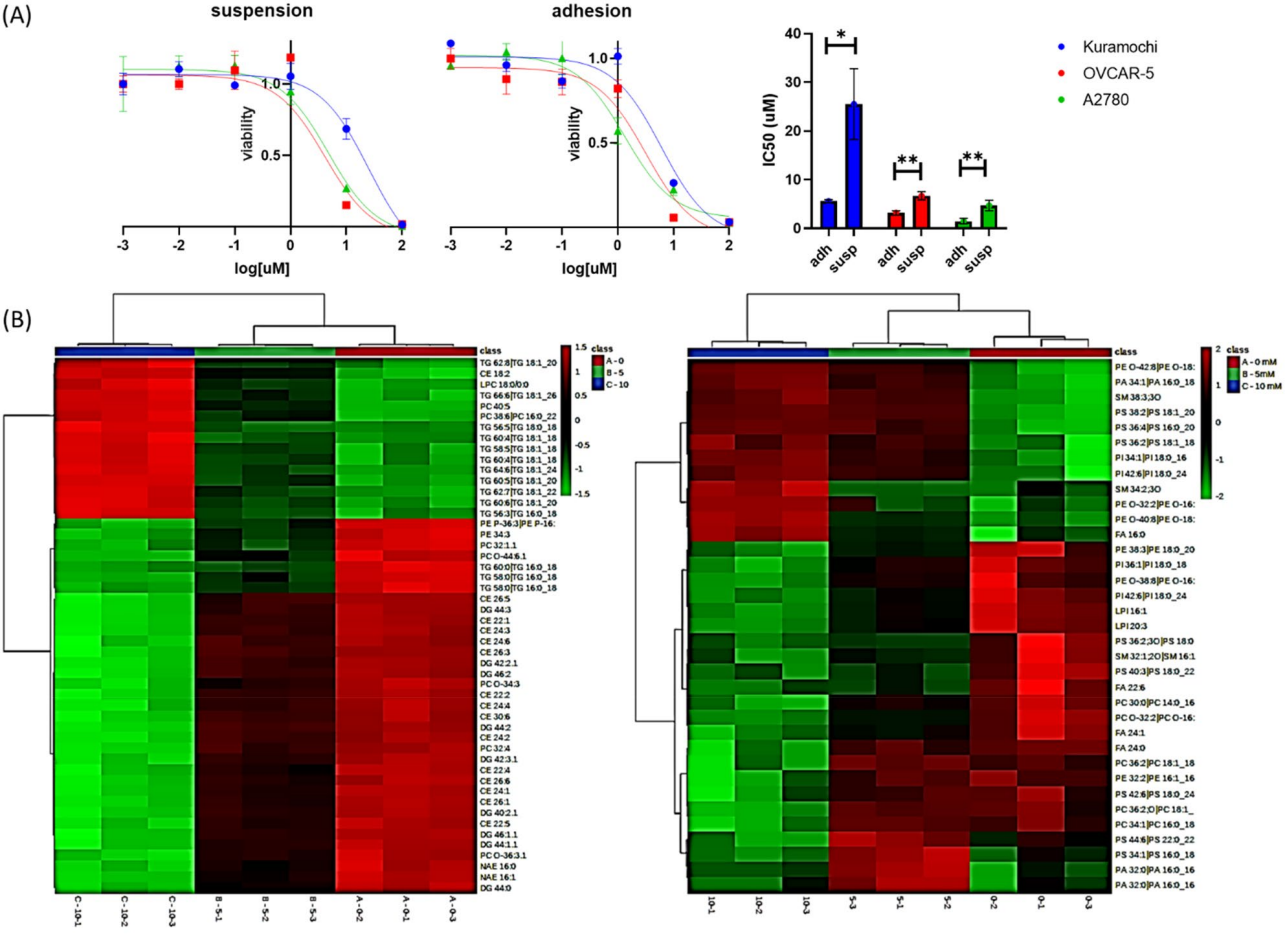
Amiodarone cytotoxicity and lipidomics analysis. (**A**) IC50 of amiodarone. On the left concentration-viability plot and fitted curves used for IC50 calculation. On the right histogram of IC50 values (t test,* p<0.05, **p<0.01). (**B**) Lipidomics analysis: heatmaps of significantly altered lipids of amiodarone treated cells. Altered lipids revealed by positive and negative acquisition mode, respectively on the right and left side. (Used acronyms: triglycerides (TG), diglycerides (DG), cholesterol ester (CE), fatty acid (FA), Phosphatidylserine (PS), phosphatidylcholine (PC), sphingomyelin (SM), phosphatidylethanolamine (PE) phosphatidylinositol (PI), phosphatidylglycerol (PG), phosphatidic acid (PA), N-acylethanolamides (NAE)).

The aging of samples resulted in a slight overall increase in size and a decrease in PdI. This is more probably due to solvent swelling than to coalescence since the average diameter is only slightly increased, proving the efficacy of PEGylation to avoid this phenomenon. Comparing liposomes produced at different FRR, smaller DFL i.e. produced with FRR3:1 and FRR4:1 show a more significant enlargement and PdI drop (under the quality threshold of 0.2) compared to FRR2:1. Aging of DFL obtained at different temperature determined the same alteration trends observed for FRR. The sample that benefited the most from storage is FRR3:1, 63_RT. Indeed, it experienced a slight increase in size but achieved a significant decrease in PdI (Fig. 5S).

### Microfluidic production of amiodarone lipidic particles ALP

Amiodarone being a practically insoluble and a hydrophobic drug (Table 3S) is suitable for passive loading and therefore to be mixed together with lipids (i.e. in the alcoholic solution) during liposome formation^34^.

Firstly, different amiodarone concentrations, ranging from 0.5 to 20 mM were tested with the selected parameters established in DFL studies (FRR3:1, TFR 1 ml/min, 63_RT, lipids concentration 10 mM). The selected amiodarone concentrations were 5 and 10 mM, indeed at higher concentration microfluidic instability occurred. Both samples appeared as milky solutions, just after microfluidic production (these solutions are named as amiodarone lipidic particles (ALP)) but the reported separation protocol allowed to obtain clear solution of amiodarone liposomes (AL) and milky solution of amiodarone particles (AP) (see method section, Fig. 7).

#### FRR effects over ALP

**Figure 6:**
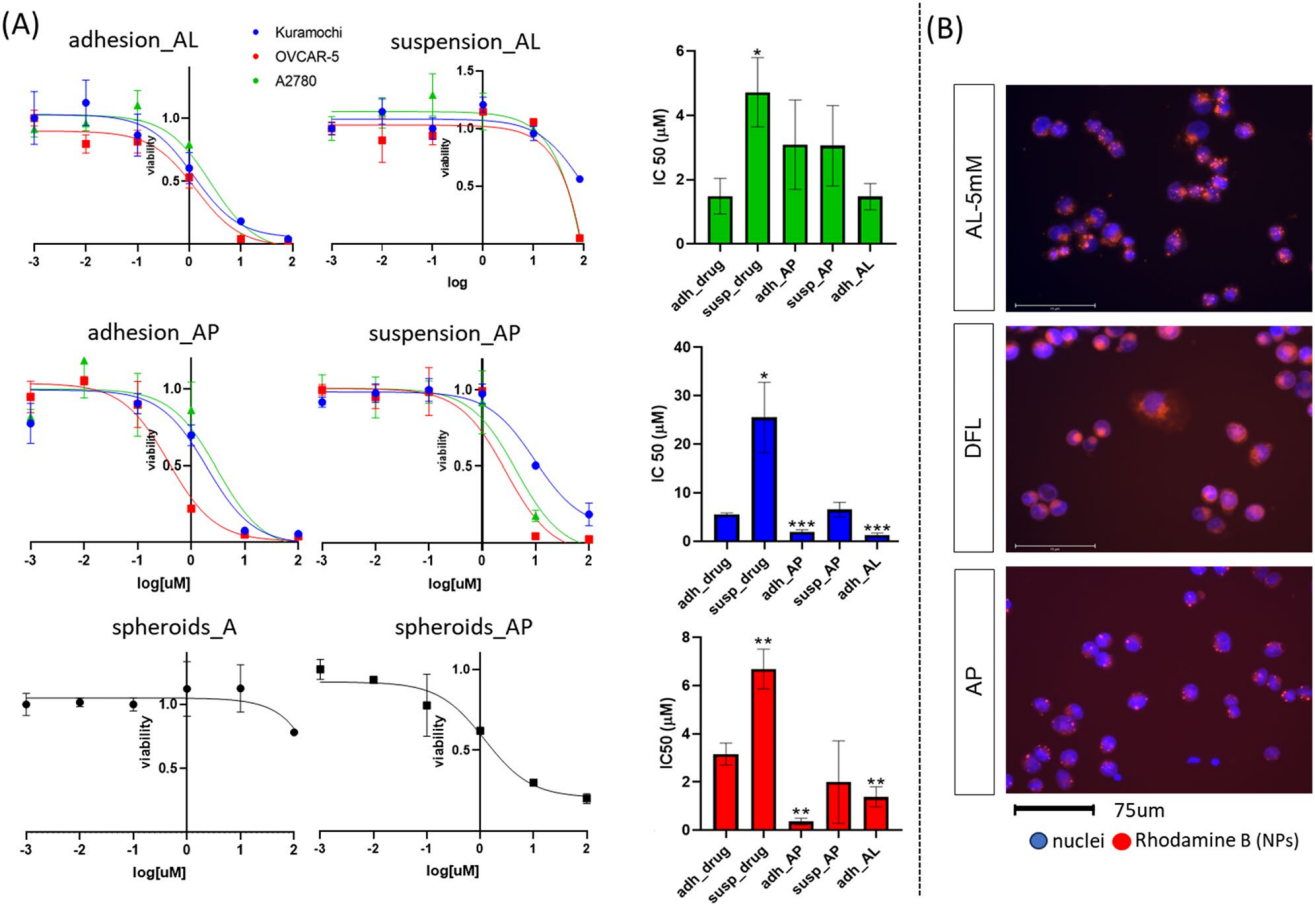
Cytotoxicity of ALP formulations. (**A**) Concentration-viability plot of AL and AP and corresponding bar plot of IC50 values (IC50 of liposomes in suspension are not reported, reported errors refer to SD). (**B**) Fluorescence microscopy images of A2780 uptake of AP, AL (amiodarone 5mM) and DFL labelled with rhodamine B.

**Figure 7:**
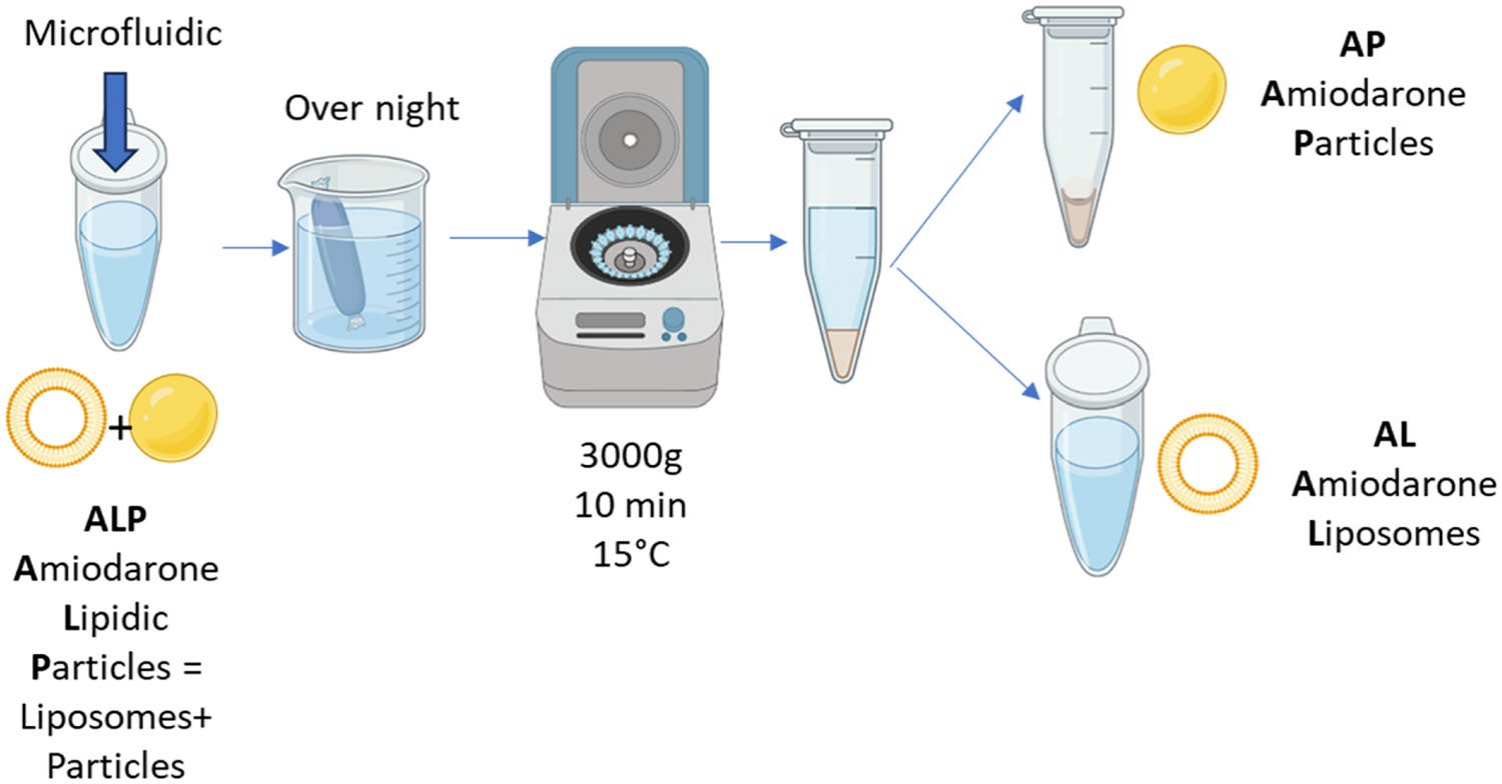
Graphical representation of ALP separation protocol.

The effect of FRR on ALP has been tested considering the results obtained for DFL and the tested different concentrations of amiodarone, therefore production parameters were TFR 1 ml/min, 63_RT, lipids concentration 10 mM, amiodarone 5 and 10 mM, FRR 2:1 and 3:1. TEM of AL samples showed that they are all composed of liposomes, while milky solutions (AP) are made by nanoparticles (NPs) which strongly tend to aggregate (Fig. 2). As expected, liposomes obtained with FFR2:1 are bigger than the FRR3:1 ones (Fig. 3, Fig. 6S). The NPs aggregation was proven to occur not only on TEM grid but also in solution, as demonstrated by DLS, and it persisted after dilution (Fig. 7S). Doxil formulation is maintained for all the AL samples, as obtained for DFL (Table 4S). Drug to lipid ratio (DL%) is significantly higher for FFR3:1 compared to FRR2:1 at both 5 mM and 10 mM amiodarone concentrations reaching about 16 mol% in FRR3:1 compared to 1 mol% of FRR2:1, considering 5 mM amiodarone concentration, it corresponds to loading capacity (LC%) of 13 ± 5% and 0.6 ± 0.3% respectively for FRR3:1 and FRR2:1. AP resulted to be constituted mainly by amiodarone, indeed (DL%) is above one thousand for all the considered conditions, with LC% values ranging from 96 ± 27% to 97 ± 10%. The present lipids are mainly cholesterol (around 98%) and traces of pegylated phosphocholine. AP characteristics were not significantly influenced by tested conditions (Table 4S).

Yields of lipids did not change significantly among the different AL samples showing a comprehensive yield around 60% (Table 5S). However, it is significantly reduced compared to DFL, so drug free liposomes, that showed a nearly total conversion of lipids. This loss of lipids is not filled considering also lipids in AP where the total lipids yield ranges from 1 to 4%. Purification step required to separate AL and AP could be a possible source of lipid loss. Encapsulation efficacy (EE%) in AL significantly differs between FRR2:1 and FRR3:1, likewise amiodarone content, with higher value for FRR3:1 (around 20%). AP samples have higher EE%, ranging from 60 to 84%, which demonstrate the major entrapment of the drug in their structure, with opposite trend between FRR2:1 and FRR3:1 compared to liposomes. Moreover, yields in AP are slightly higher for 10 mM compared to 5 mM amiodarone concentration, indeed 5 mM samples required longer time before presenting noticeable milky appearance.

#### Temperature effect over ALP

Considering the above results, FRR3:1 was used to evaluate temperature effects over both AL and AP (total flow rate of 1 ml/min, lipid concentration 10 mM).

Reproducibility of AL was not affected by different temperatures of reservoirs, differently from what has been determined for AP, which showed a good reproducibility only for 63_RT (Fig. 8S). Moreover, the temperature differences are not altering AL average diameters when 5 mM of amiodarone is used. However, for 10 mM of amiodarone at RT_RT, larger liposomes are obtained, contrary to what resulted for DFL (Fig. 1). The PdI of AL meets quality requirements, differently from what reported for DFL, and it maintained Doxil composition (Table 6S). In AL samples, drug to lipid ratio (DL) % and EE% are lower for 63_63 than the other two temperature conditions at both 5 mM and 10 mM of amiodarone (Tables 6S and 7S). The maximum EE% was equal to 21 ± 5% while le lowest 3 ± 1%, obtained respectively for 63_RT 5 mM and 63_63 10 mM.

DLS analysis of AP confirmed the strong tendency of NPs towards aggregation for all the samples. 63_RT with 10 mM of amiodarone showed smaller diameter and PdI, being below the quality threshold (Fig. 1). Composition of AP samples showed few significative differences among different conditions, verifying what previously reported in FRR study; NPs are mainly made by amiodarone with cholesterol and traces of pegylated phosphocholine (Tables 8S, 9S).

#### Stability over time of ALP

The ageing of AL and AP was studied with samples obtained with production conditions selected basing on previously reported studies (FRR3, 63_RT, lipid concentration 10 mM). AL showed a little increment of diameter paired with PdI stabilisation over time (Fig. 9S). While, ageing of AP caused the increment of PdI for sample obtained with 10 mM of amiodarone paired with stable size. In general, it can be said that both AL and AP are stable after one month of storage with no sign of aggregation occurring in both samples.

#### Comparison between DFL and ALP

Optimised conditions were used to produce DLF, AL and AP (total flow rate 1 ml/min, FRR3:1, 63_RT, 10 mM of total lipids). Amiodarone was used at 5 and 10 mM, and reported for all the samples later described.

Z potential of DFL, AL and AP is around − 2 (Table 1) therefore the encapsulation of amiodarone is not altering the charge of the particles.

**Table 1:** Zeta potential values of different samples of AP and AL produced with 5 and 10mM of Amiodarone, FRR3:1, total flow rate = 1ml/min, 63_RT, lipids 10 mM.

Amiodarone(mM)	NPs	Zeta potential (mV)
5	AL	− 2.0 ± 0.4
10	AL	− 1 ± 1
5	AP	− 2 ± 1
10	AP	− 2.8 ± 0.6

In order to check if any crystals of the drug were formed into particles, x-ray diffraction analysis were recorded proving the amorphous state of all the particles components. Indeed, particles spectra showed only peaks correspondent to phosphate buffer saline (PBS), hence deriving from the drying on the sample holder (Fig. 3).

The atomic force microscopy (AFM) analysis of DFL, AL and AP (produced with 5 mM amiodarone) revealed the presence of roundish particles with heights ranging from 3–4 nm to 50 nm for liposomal samples while in the case of AP also the presence of membrane patches (3–4 nm in height) and NPs aggregates was proved (Fig. 10S). The analysis of height-diameter correlation showed that AL has a height on average higher than DFL, sign of higher rigidity, possibly due to the drug encapsulated (Fig. 3). For the AP sample, the height-diameter correlation is less accurate due to the not homogeneous shape of the particles and the aggregates were observed.

The higher rigidity of AL is confirmed even by the comparison between DLS and TEM determined sizes. Indeed, DFL and AL produced at FRR2:1 i.e. liposomes without amiodarone or with low encapsulation, showed bigger average size for TEM than DLS, while it is the contrary for AL with drug to lipid ratio of 16 (FRR3:1) (Figs. 1, 3). Therefore, in the case of the absence or low content of amiodarone, liposomes exhibit enhanced deformation during TEM grid preparation, likely due to the higher elasticity of the membrane.

#### Study of colloidal behaviour of amiodarone

Amiodarone mixing in aqueous solution, both through microfluidics and simple mixing, promoted the formation of milky solutions, with opalescence decreasing during time until a clear solution was obtained about 12 h later. DLS analysis over time of the solution obtained by flowing 5 mM amiodarone into microfluidic revealed the presence of a micrometric peak, which however decreased in size during time and finally disappeared in about 30 h (Fig. 3). Optical microscope examination of the solution confirmed the presence of particles with micrometric size.

### Comparison of CPT1A expression and metabolic FTIR profile of adhesion and suspension cultures

To evaluate if the CPT1A was overexpressed in suspension cultures, western blot analysis was performed. Western blot results revealed that OVCAR-5 has higher levels of CPT1A compared to A2780 and Kuramochi. Comparing the two different cultures, a modest increase of expression of CPT1A for suspension culture was determined with OVCAR-5 and Kuramochi showing a more consistent increment (Fig. 4). Fourier transformed infrared spectroscopy (FTIR) analysis revealed significantly different profile for adhesion and suspension cultures as highlighted by principal component analysis (PCA) and hierarchical cluster analysis (Fig. 12S) mainly drive by three spectral areas: lipid region (2800–2950 cm^−1^), peak shoulder assigned to fatty acid ester /phospholipids (1730–1750 cm^−1^), first band in the area of methylene and methine bending assigned to fatty acids and side chain of amino acid (1450–1470 cm^−1^) (Fig. 4). Moreover, integration of lipid peaks (2800–3000 cm^−1^) confirmed significantly higher values for suspension cultures. Therefore, the spectral areas that are mainly responsible of differences between adhesion and suspension cultures can be all related to lipids.

### Amiodarone effects in ovarian cancer cell lines

Amiodarone cytotoxicity was evaluated both in adhesion and suspension cultures obtaining IC50 values ranging respectively from 1.5 to 5.6 and from 4.7 to 25 μM (Fig. 5A). IC50 results obtained for adhesion cells are in line with a previous study^21^ and analogue to the values obtained for cisplatin, a traditionally applied drug in ovarian cancer treatment (Table 10S).

#### Alteration of cellular lipidome induced by AM treatment

To evaluate if amiodarone acts as an inhibitor of CPT1A and therefore influence lipids, lipidomics analysis was performed on A2780 cultured in adhesion treated for 96 h with the drug at concentration equal to 5 and 10 µM. Given that cells were grown on critical conditions, namely high concentration of amiodarone, we decided to move from FTIR to mass spectrometry analysis, in order to avoid misclassification of IR spectra alterations. Amiodarone determined a significant alteration of the lipidome of cells (Fig. 5), showing clearly separate groups in PCA obtained for both positive and negative acquisition mode (Fig. 13S). Heatmaps show a general triglycerides (TG) increment associated with the increase of amiodarone treatment, and an alteration of long chain fatty acids, which however differs among different species with increasing level of fatty acid 16:0 and decrease of 22:6, 24:0, 24:1. Moreover, drug treatment determined a decrease of cholesterol esters and a various alteration of phospholipids.

#### Amiodarone and doxorubicin synergism

Doxorubicin was selected to be tested in combination with amiodarone, being a widely applied chemotherapeutic agent and considering the previously discussed clinical trial, which involved its administration in combination with amiodarone. The median-drug effect analysis method is the most used way to establish the efficiency of drugs combination. The fit of data into the media effect plot, also known as Chou Talalay (CT) plot, lead to calculate combination index (CI) value that allows a quantitative definition of synergism (CI < 1), antagonism (CI > 1) and additive (CI = 1) effects. Another parameter defined by CT equation is dose-reduction index (DRI) and it indicates, for a fixed effect level, how many folds the dose of each drug may be reduced when it is used in a combination compared with the drug alone^35^. The dose reduction is favorable for DRI above 1, not favorable for DRI below 1 and DRI equal to 1 indicates no dose-reduction (Chou and Talalay^36^). Both CI and DRI are reported as a function of fraction affected (Fa), which is a measure of cells mortality rate (Fig. 14S). The goodness of the CT plot fit was confirmed by linear correlation coefficient (r) which was above 0.95. Amiodarone combination with doxorubicin corresponds to CI below 1 for Fa values up to 0.35. Synergism is also visible from logarithmic plot of CI vs Fa, which condenses the graph allowing to visualize also points out of scale, and demonstrates synergism (logCI < 0) for low Fa values. DRI values likewise demonstrate favorable doses reduction (log DRI > 0) for Fa values up to 0.65 confirming synergism of the two combined drugs.

### Cytotoxicity of ALP

Basing on previously reported results for ALP, production conditions of FFR3:1, amiodarone 5 mM, and temperature 63_RT were chosen for in vitro experiments (TFR = 1 ml/ml, 10 mM lipid concentration). The AP formulation in suspension cultures allowed to obtain IC50 values comparable to the ones obtained for the free drug in adhesion cultures. AL were active in adhesion culture, where both formulations decreased the IC50 values for Kuramochi and OVCAR-5, while IC50s were comparable to the one of the free drug for A2780. AL in suspensions demonstrated the lowest activities of all the tested treatments (Fig. 6). SKOV3 are known to form spheroids when forced to grow in suspension, hence it can represent a good model to evaluate the permeability of AP. Therefore, AP were tested on SKOV3 spheroids obtaining an IC50 value equal to 2 ± 1μM comparable with the one obtained for the other cell lines, while the free drug seems to be ineffective (Fig. 6). Internalization of DFL, AL, AP was evaluated by incubation of A2780 treated by fluorescently labelled particles. All the tested particles have been proved to be internalized by cells given the red fluorescence coming from the cytosolic compartment of cells (Figs. 6).

## Discussion

In the current work the effects of temperature and FRR over DFL production were studied^29,30^. In contrast with literature^28^, we proved the possibility to obtain single population of SUV DFL (rigid lipid mixture) even at room temperature while, with this formulation, LUV are probably formed as results of high lipids concentration. Data regarding liposome production through microfluidics are important since mechanisms of their formation in the chip are still debated but essential to understand and optimize drug encapsulation.

For ALP, the production of liposomes containing amiodarone with maximised loading of the drug was accompanied with the formation of NPs with strong tendency toward aggregation that was proved, beside by TEM images, also analysing liquid samples both with DLS and AFM. The nano-components of the aggregates might be defined as micelles basing on their TEM appearance in term of interaction with the staining^37,38^ but also considering the colloidal behaviour of amiodarone in water at low concentration^39,40^. However, micelles are well known for being unstable and dissolve by dilution^41^ while aggregates in AP samples showed stability over dilution. Furthermore, Benedini et al. established that the amiodarone Krafft temperature (TK), is equal to 70.6 °C. Therefore, the milky appearance of the solutions below 70 °C cannot be attributed to micelle formation. Given the above considerations, the nano sized objects obtained in the pellet were termed amiodarone particles (AP). In order to indagate AP formation, amiodarone colloidal behaviour was studied demonstrating that the drug addition to water, both in presence or absence of lipids, generate milky solutions. However, the nature of the obtained solutions is different, indeed in absence of lipids the obtained colloids are not stable. Coacervate were reported in literature^40^ as an explanation for milky opalescent solution obtained by amiodarone mixing with water (0.5–8 mg/ml) heated to 60 °C and then equilibrate to room temperature^40^. In the present study, the hydrodynamic diameters of amiodaronewater solutions in absence of lipids are in line with the results of Bouligand et al. Therefore, it can be assumed that coacervates formation are responsible for the milky appearance of lipids free amiodarone water solutions. Coacervate, during time, tend to produce crystals^39^, in line with the disappear of DLS peak observed in the present study. Differently, AP were proven by DLS to be stable even after 1 month and without formation of crystals as confirmed by XRD analysis. The AP stability and the high amiodarone content of AL may derive from the high affinity of the drug for membrane lipids, that are well represented by HEPC and cholesterol. Indeed, previous studies indagated the behaviour of amiodarone in phospholipids membrane establishing that amiodarone effect depends upon cholesterol concentration, which in turn defines membrane liquid phases^42^. Above cholesterol 30%mol, amiodarone promotes membrane disorder since it combines with cholesterol producing a cylindrical structure that can be stabilized into phosphocholine bilayer^43^increasing the disorder^42^. Instead, even if cholesterol was present in high percentage, in the present study amiodarone seems harden liposomes vesicles therefore corresponding to more ordered bilayers. However, it has to be considered that liposomes have been forced to form in the micromixer at different temperatures, amiodarone concentration and lipids compared to previous studies. Despite this difference, amiodarone tendency to form cylindrical structure with cholesterol is established and can be responsible also for the formation of membrane patches and others obtained structures^44^.

The amiodarone encapsulation into AL was particularly successful at FRR3:1, reaching drug to lipid ratio value about 15% w/w (which corresponds to 16 mol%) analogues to doxorubicin amount in Doxil formulation (12% w/w)^45^. The drug encapsulation did not negatively affect liposomal size and distribution and neither Doxil lipid composition. However, the yields of lipids forming liposomes sensitively decrease as effect of the simultaneous production of AP. Moreover, the loading of the drug change elasticity of liposome membranes as highlighted through AFM analysis and supported by DLS and TEM size determination. The higher rigidity suggests that the amiodarone is encapsulated into the bilayer but at the same time it can’t be excluded that the high loading capacity is obtained by the entrapment of few nanoparticles of AP into the aqueous core increasing the rigidity.

DrugPredict is a computational drug-repositioning system which was used for exploring FDA approved drugs as novel candidates for drug repositioning in epithelial ovarian cancer. Amiodarone was ranked within the 3.9% of potential drugs which is a significative result considering that one of the most used chemotherapeutic agents, namely carboplatin, reached the 3.7%^21^. Therefore, amiodarone represents a promising drug for the treatment of epithelial ovarian cancer.

The present study confirmed the in vitro activity as anticancer drug of amiodarone and highlighed synergism with doxorubicin. However, synergism occurred at low doses which correspond to low Fa, namely low mortality rate. Synergism is not equally important at all drug concentrations and in case of cancer treatment, it would be more promising if it appears at high concentratinos which correspond to an almost complete suppression of cells (Fa ≈97%), which could better delay or avoid cancer relapsing^46^.

The present study confirmed the in vitro activity as anticancer drug also for anoikis resistant cells, which are considered to overexpress CPT1A and increase lipid metabolism. Even if modest, an increase of CPT1A in suspension cultures was determined for all the cell lines, in particular OVCAR-5 and Kuramochi. This can be explained considering that both OVCAR-5 and Kuramochi are representative of high-grade histological subtypes, which are more prone to disseminate in the peritoneal cavity. FTIR results, combined with western blot analysis, seems to proof the presence of the metabolic shift in suspension condition, revealing an increment in lipid peak areas. This result is in agreement with a previous study evaluating the fatty acids content of adhesion and suspension cells (48 h), which determined an accumulation of long- and very-long chain fatty acids in anoikis-escaped cells^8^. Also, amiodarone treated A2780 cells showed clear lipid alterations. In particular, the increase of triglycerides levels can be a consequence of the block of fatty acids transport into the mitochondrial matrix determined by the inhibition of CPT1A^47^. Moreover, amiodarone treatment is reported to cause phospholipidosis, namely the excessive phospholipid accumulation in the lysosomes of cells^48^. Therefore, the observed alteration of phospholipids may derive from this phenomenon, even though different species show opposite trend. Interestingly palmitic acid concentration increases in amiodarone treated cells, while longer and unsaturated fatty acids decrease, whose increase is instead associated with anoikis resistance^8^. The accumulation of palmitic acid could be the direct effect of CPT1A inhibition, which however has different effects on short and long fatty acids^47^.

The efficacy of amiodarone in cell viability reduction was confirmed from IC50 values for adhesion cultures being close to the one obtained with cisplatin. However, even if amiodarone has an inhibitory activity on CPT1A, its efficacy in cell viability reduction in suspension cultures is lower. Considering that cisplatin maintained its efficacy in suspension, the decrease in the efficacy of amiodarone might be attributed to its low solubility. Indeed, low solubility can be a limiting factor reducing the diffusion into cell aggregates, which form in suspension condition. Amiodarone low aqueous solubility represents a limit also for the drug administration, requiring for injectable formulations the addition of surfactants and co-solvent. Marketed formulations are compounded with amiodarone hydrochloride (50 mg/ml), polysorbate 80 (Tween 80, 100 mg/ml) and benzyl alcohol (20 mg/ml) generally in 3 mL ampuls. Other strategies reported in literature proposed the formulation of amiodarone in aqueous media through the dispersion of the hydrochloride salt 0.1 mol/l in pH = 3.8 acetate buffer^49–51^. Excipients are reported to contribute to the manifestation of side effects such as inotropy and hypotension^52^. Thus, increase amiodarone solubility is a challenge that still need to be addressed and it could represent an important improvement for its administration. In this work, amiodarone drug delivery system enabling to overcome the low solubility of the drug i.e. AL and AP, stable nano formulations were tested over epithelial ovarian cancer models. It has to be noted that ALP can be used to obtain formulations at higher concentration of amiodarone than the ones applied in the present study, since they can be isolated by centrifugation and redisperse in a volume of solution which fits the purpose of their application, without requiring the addition of excipients that further compromise the toxicity of amiodarone administration. AL obtained with 5 mM of amiodarone and FRR1:3 were selected to be tested in vitro given the high drug to lipid ratio. However, AL resulted effective in adhesion cultures but showed a reduced activity in suspension. Instead, AP was efficient also for suspension cultures and for SKOV-3 spheroids. Moreover, in adhesion culture both AL and AP resulted to be internalized by cells. This seems to suggest that the minor efficacy of the drug in suspension cultures is determined by the low ability of the drug to diffuse into cells aggregates, while AP formulation may overcome the penetration limit. AL and AP could be suitable for different amiodarone administrations. Liposomes are optimized for intravenous injection, followed by enhance permeation and retention effect accumulation in cancer site. While, a possible administration of AP, being present in the form of aggregates, could be the intraperitoneal injection. Intraperitoneal injection has been identified as a promising way for target epithelial ovarian cancer metastasis however, the high clearance rate constitutes a limit^53,54^. AP aggregates might act as depot of the drug allowing a prolonged release of the drug^55^. Moreover, the amorphous state of the drug in both formulations would allow a higher dissolution from the drug delivery systems compared to crystal/nano-crystal phase^56^.

## Conclusion

Amiodarone was encapsulated into ALP trough microfluidics obtaining simultaneously liposomes with high drug content (drug to lipid ratio 15%) and NPs mainly composed by the drug. Production parameters effects over DFL and ALP were tested, and all the particles were fully characterized. Optimized formulations were used to test amiodarone activity over epithelial ovarian cancer models. The use of computational methods could be useful to precisely individuate drug localization into AL.

The role of CPT1A in the amiodarone efficacy over epithelial ovarian cancer models was investigated. CPT1A was proven by western blot to be overexpressed in suspension cultures of A2780, Kuramochi, OVCAR-5 even if the increment is only determinable after densitometry analysis since the fold increase is modest. This result is also confirmed by FT-IR, which revealed a significative different profile for adhesion and suspension culture with lipids peaks being the main responsible of the difference. The amiodarone resulted efficient in reducing cell viability, however its effect is lower in suspension condition. Lipidomics profile of treated and not treated cells revealed a significant alteration of lipid content in treated cells which is in line with the inhibition of CPT1A. However, in order to better evaluate amiodarone CPT1A inhibition features the evaluation of fatty acid oxidation rate, such as by SeaHorse Flux Analyzer, would be useful.

The reduced ability of amiodarone in suspension cultures could be determined by cell aggregate resistance to drug diffusion. AP has been proven to be particularly useful to restore amiodarone effect in suspension cultures and hence overcome diffusion limit, while liposomes were effective only in adhesion culture. However, these two formulations could be suitable for different administration routes. In *vivo studies* would be useful to fully understand the potential clinical used of these amiodarone drug delivery systems.

## Materials and methods

### Chemicals

Lipids were bought from Avanti Polar Lipids: HEPC (Egg, Chicken, 840,059); CHO (700,000); DSPE-PEG (880,120). Materials bought from Merck: Dulbecco′s Phosphate Buffered Saline (DPBS), (D1408); AM (A8423); deuterated chloroform (151,823); benzoic acid (TraceCERT); penicillin–streptomycin (P4333). Materials bought from Sigma Aldrich: Poly (2-hydroxyethyl methacrylate) (Poly-HEMA) (P3932); Ethanol (V001229); sodium orthovanadate (S6508); sodium fluoride (S6776); NaCl (S9888); rhodamine B 10 mg/ml (83,689). Cell culture media RPMI 1640 and trypsin (25,200,072) were acquired from ThermoFisher Gibco, while Hoechst (33,342) was bought from Thermo Fisher Scientific. Fetal bovine serum (FBS) was taken from Microtech (Napoli,Italy). Protease Inhibitor Cocktail was bought from Roche (04,693,116,001), secondary antibodies from Invitrogen (Goat anti-Rabbit 1gG 32,460, Rabbit anti- Goat IgG 31,402), CPT1A (D3B3) Rabbit mAb from Cell Signaling Technology (12,252) and vinculin Goat mAb from Santa Cruz (sc-7649). Protein Assay Dye was taken from Bio-Rad (5,000,006), Sample buffer 1X from GenScript (MB01015) and LiteAblot^®^ EXTEND Chemiluminescent Substrate from Euroclone (EMP013001). Water was purified with Milli-Q^®^ (Millipak^®^ 0.22 μm) system.

### Microfluidic synthesis

To produce both empty liposomes based on Doxil® formulation (DFL) and amiodarone lipidic particles (ALP) was applied a microfluidic set up composed by commercially available components: microfluidic apparatus (Fluigent, Okabé, France) constitute of two pressure driven pumps (7 bar Flow EZ), two flow controllers (flow unit XL); coupled with a staggered herringbone micromixer glass chip (HMC) (Darwin Microfluidics, Paris, France). The microfluidic apparatus was controlled by the Microfluidic Automation Tool software which allows to create protocols and automated experiments.

Doxil formulation was used to produce both Doxil formulated liposomes (DFL) and ALP i.e. HEPC:CHO:DSPE-PEG 55:40:5 as molar ratio^30^.The lipid mixture was dissolved in ethanol, together with amiodarone for ALP, and mixed in the chip with Dulbecco's Phosphate-Buffered Saline DPBS. Basing on previous reported^28^ and verified optimised conditions a total lipids concentration of 10 mM (equal to 7.2 mg/ml) and a total flow rate of 1 ml/min (sum of the flow rate of ethanol and DPBS solutions streams) were used. Different flow rate ratios (FRR, ratio of the flow rate of DPBS solution to ethanol solution) were tested together with different temperature conditions. In the case of ALP also different amiodarone concentrations were explored (details reported in the results section).

Samples collected from microfluidics were dialyzed overnight in DPBS by Slide-A-Lyzer™ MINI Dialysis Devices, 20 K MWCO (88,405, ThermoFisher) to remove all the free molecules and ethanol to avoid interdigitation of lipids in the bilayer of liposomes.

In this work, to establish a liposomes production protocol that might be useful for temperature sensitive drugs, three different temperature conditions were tested: 63_63 (both reservoirs heated at 63 °C), 63_RT (heating the alcoholic reservoir) and RT_RT (both reservoirs unheated). Temperature of heated solutions was determined considering the requirement of operating 10 °C above the melting points (Tm) of lipids mixture to avoid gel phase interference in the process of vesicles self-assemblies^28^ and since Doxil^®^ formulation has Tm = 53 °C^30^.

### Separation protocol

For ALP, a separation protocol followed the dialysis of samples Fig. 7. Samples were centrifuged by Micro Star 17R (Avantor, Radnor, Pennsylvania, USA) (3000 g, 10 min, 15 °C). Both the obtained pellet (AP) and supernatant (AL) were resuspended in DPBS and washed with DPBS by repeating three times the centrifugation in DPBS.

### H-NMR for particle composition analysis

Samples were processed as described above then AL and DFL were ultra-centrifuged at 200,000 *g*, 15 °C for two hours with Sorvall™ WX + Ultracentrifuge Series (Thermo Scientific, Waltham, Massachusetts). Ultracentrifuged DFL and AL pellets along with centrifuged pellet of AP (3000 *g*, 10 min, 15 °C) were frozen at − 80 °C, then lyophilized with LIO5P (Cinquepascal S.R.L., Milan, Italy). The dried samples were resuspended in 600 µL of deuterated chloroform. Before transferring the chloroform into NMR tubes, samples have been vortexed 30 s, bath sonicated for ten minutes, vortexed again 30 s and finally centrifuged 10000 *g* for 2 min. When lipids quantification was required, a known amount of benzoic acid was added to samples solutions.

Data were acquired by Bruker 400 MHz Advance spectrometer (Bruker, Billerica, Massachusetts). Acquisition parameters consisted in a 20.02 ppm spectral width, 30° pulse, 4.08 s acquisition time, 1 s relaxation delay, 64,000 data points, 64 scans. MestReNova^®^ (Mestrelab Research, Santiago de Compostela, Spagna) software was used for processing proton free induction decay data typically by Fourier transformation, phasing, referenced to the low levels of protonated chloroform.

Lipid concentrations were calculated from external standard calibration curves. Peaks assignment is reported in Fig. 1S and Table 1S.

From H-NMR analysis drug over lipids ratio (DL%), loading capacity (LC%) and encapsulation efficacy (EE%) were calculated as follow:

$$DL\%=100*\frac{ entrapped\, amiodarone \,(mol) }{total\, lipids\, (mol)}$$

$$LC\%=100*\frac{weight \,of \,entrapped\, amiodarone}{total \,particles \,weight}$$

$$EE\%=\frac{entrapped\, amiodarone}{added\, amiodarone}. 100$$

H-NMR was used to calculate also yield of lipid conversion into lipidic particles (EE% corresponds to amiodarone yield) and their composition expressed as mol% on total lipids (DL% corresponds to amiodarone composition).

To test H-NMR method reproducibility, a pooled sample of DFL was divided into three parts which were individually subjected to preanalytical protocol and measured for quantification.

### X-ray diffraction (XRD)

XRD was performed to study the crystalline nature of the formulations. Amiodarone powder was measured using powder specimen holder, while amiodarone particles, amiodarone liposomes and PBS were deposited by repetitive steps of dripping and drying into Si low background sample holder until a visible film was obtained on the surface of the holder.Samples were analysed by XRD diffractometer Empyrean (Malvern Panalytical, Worcestershire, UK). CuKα radiation was used, the diffractograms were recorded by setting a diffracted intensity detection step equal to 0.05°. The unit of measurement of the detected intensity is the number of pulses / second. The XRD spectra reported the intensities of the diffracted X ray beams as a function of the angle.

### Dynamic light scattering (DLS)

DFL, AP, AL, zeta potential, hydrodynamic diameters and distribution (polydispersity index PdI) in PBS were determined by Zetasizer Nano particle analyser (Malvern Panalytical, Malvern, UK).

### Stability over time

DFL, AL, AP samples deterioration over storage was evaluated by DLS analysis over time up to one month. Samples were dispersed in DPBS and maintained at 4 °C.

### Transmission electron microscopy (TEM)

A drop of solution (about 25 μL) was deposited on a 400-mesh holey film grid; after staining with 1% uranyl acetate (2 min), the sample was observed with a FEI Tecnai G2 transmission electron microscope operating at 100 kV (Hillsboro, Oregon, US). The images were taken with a Veleta digital camera (Olympus Soft Imaging System). Image processing was carried out by ImageJ software^57^.

### Atomic force microscopy (AFM)

Samples were deposited on poly-ornithine functionalized mica. For the deposition 20 µL of particles solution were left on the mica surface for one hour and then gently rinsed with PBS. The samples were not dried and a drop of PBS solution was always kept on the surface. The measurements were carried out in PBS with an MFP-3D Asylum research instrument in dynamic mode using soft cantilevers (BLAC40- TS Olympus, radius of curvature < 10 nm, spring constant 0.1 N/m). Images were acquired with 8 nm pixel size at 0.75 Hz scan rate in at least 5 different positions on the sample.

### Study of colloidal behaviour of amiodarone

5 mM and 10 mM solutions of amiodarone in ethanol were flowed into the microfluidic device in the same conditions used for ALP synthesis. Moreover, amiodarone solution 10 mM, was mixed with PBS in the same ratio of FRR3:1 both at room temperature and after being heated at 60 °C, in analogy with microfluidic protocol.

### Cell culture

Human ovarian cancer cell lines A2780 (Sigma (Inc., St. Louis, MO, USA), Kuramochi (JCRB Cell Bank), OVCAR-5 and SKOV3 (gently provided by Dr. Gustavo Baldassarre), were cultured in RPMI 1640 media supplemented by 10% v/v FBS and penicillin–streptomycin 1% maintained at 37 °C in a humidified atmosphere containing 5% CO_2_ according to the supplier. Cell splitting was performed at 70–80% of confluency using trypsin. The morphology and growth of cells were monitored daily under a microscope to ensure their healthy status. Suspension cultures, which are models of anoikis resisting cells, were obtained coating plates with Poly-HEMA (Sigma Aldrich, P3932) solution of 5 mg/ml in ethanol 96%. Suspension culture of SKOV3 cell line allowed to test drug efficacy on spheroids giving the spontaneous formation of these structures in suspension culture. Therefore, SKOV3 spheroids are a good model for ovarian cancer metastasis but also for penetration evaluation.

### Western blot

Three independent cultures for each considered condition were grown for 48 h into 10 cm plate (seeding density 2 × 10^6^). Adhesion cells were collected using a scraper, all the pellets were washed two times with DPBS by centrifugation at 1500 *rpm* for 5 min (Multifuge™ X1, Thermo Fischer, Waltham, Massachusetts) to remove traces of medium and kept on ice. Samples were then lysed using RIPA buffer (50 mM Tris-HCI pH 8.0, 150 mM NaCI, 1% IGEPAL, 0.5% sodium deoxycholate and 0.1% SDS) supplemented with Protease Inhibitor Cocktail, sodium orthovanadate and sodium fluoride. Protein concentration was estimated by Bradford assay by using Bio-Rad Protein Assay Dye. Samples were prepared using equal amounts of protein (25–40 ug), 5X Sample Buffer and diluted to a final volume of 20 uL with MQ. Samples were heated at 100 °C for 10 min and proteins were separated by 4–20% SDS–polyacrylamide gel electrophoresis (GenScript, M42012) and transferred to nitrocellulose membrane (GE Healthcare, 10600002) using Mini-PROTEAN Tetra Vertical Electrophoresis Cell (Bio-Rad, 1658004). The protein signal was detected with LiteAblot^®^ EXTEND Chemiluminescent Substrate using VWR^®^ Imager CHEMI Premium (VWR, 730-1469P). Primary antibodies are CPT1A (D3B3) Rabbit mAb and vinculin Goat mAb. The secondary antibodies used are Goat anti-Rabbit IgG and Rabbit anti- Goat IgG. Images were analysed using GelAnalyzer 19.1 (www.gelanalyzer.com, created by Istvan Lazar Jr., PhD and Istvan Lazar Sr., PhD, CSc) and average were obtained from three biological samples. Vinculin band was used for normalization purpose.

### ATR FT-IR metabolomic profile of cells

FTIR analysis of cells allows to obtain significant information on content, structures and dynamics of biomolecules such as lipids, nucleic acids, proteins and carbohydrates which have been used to establish the effects of drugs, pollutants or cell cycle stage on various cell lines both healthy and cancerous^58–61^. Therefore, differences in the biomolecules content of different culture and cell line, with a particular focus on lipids, have been evaluated by the analysis of the ATR- FTIR spectra of cell pellet samples.

Three independent cell cultures for each considered condition were grown for 48 h into 6 wells plate (seeding density 5 × 10^5^). Adhesion cells were collected using a scraper, all the pellets were washed three time with isotonic water solution NaCl 0.9% by centrifugation at 1500 *rpm* for 5 min by Micro Star 17R. Three biological replicates were obtained for each evaluated condition.

FTIR measures were performed at room temperature with Nicolet NEXUS 670 FTIR (Thermo Scientific, Waltham, Massachusetts) in ATR mode using a Zn/Se crystal. Blank was recorded before every sample and subtracted from the sample spectra. For each biological replicate 5 different spectra have been recorded given a total of 15 spectra per condition. Acquisition parameters were: 128 scan, resolution 4 cm^−1^, absorbance mode, spectral window 600- 4000 cm^−1^.

Spectra pre-processing was performed by Origin (OriginLab corporation, Northampton, Massachusetts), SpectraGriph 1.2.14 software (Optical spectroscopy software 2016–20 developed by Dr. Friedrich Menges, Oberstdorf, Germany), Excel (Microsoft Office, Redmond, Washington). Water vapour contribution has been removed by subtracting weighted spectrum of pure water vapor from sample spectra as reported by Lasch^62^. Briefly spectrum of water vapour was acquired in the same conditions of samples spectra and second derivatives of samples and water vapour spectra were calculated. Correction factor was calculated by the ratio of the second derivative intensities values of samples and water vapour. Finally, water vapor correction is carried out by subtracting from sample spectra the product of the water vapor spectrum and the correction factor. Then spectra were baseline corrected over the whole spectrum (at points 800, 904, 941, 1352, 1483, 1765,1 801, 1934, 2123, 2274, 2515, 2598, 2702, 2800,2997, 3716, 3998 cm^−1^) and normalized for amide II peak area giving a constant protein concentration. Processed spectra were used for peak integration and statistical analysis which was performed by MetaboAnalysist (web-based comprehensive platform for metabolomics data analysis, developed by Xia Lab, Montreal, Canada) after appropriate normalization, scaling and transformation of data. T test was performed in order to identify the spectral areas that mainly contribute to differentiate the FT-IR profile of adhesion and suspension culture. Given the high numbers of variables, false discovery rate (FDR) was taken into account instead of p value and spectra were considered significantly different for FDR < 0.01.

### Cell lipidomic analysis

The analysis was performed on cell pellets of adhesion culture which allow to remove death cells by PBS washing of the plate, while this separation of death cells was not possible for suspension culture. Three independent cultures of A2780 were seeded in 10 cm plates (density 0.5 × 10^6^) and 24 h later treated with 5 mM, 10 mM of amiodarone or not treated. Cells were grown for 96 h then carefully washed with PBS to remove died cells and collected using a scraper. All the pellets were washed two times with PBS by centrifugation at 1500 rpm for 5 min (Multifuge™ X1, Thermo Fischer, Waltham, Massachusetts) to remove traces of medium and kept on ice. A comprehensive cell lipidomic analysis was conducted using reversed-phase liquid chromatography-high-resolution mass spectrometry (RPLC-HRMS). The cell's lipids were extracted using a modified Folch extraction protocol and were separated using a C18 Eclipse Plus 100 × 2.1 mm column (Agilent) with mobile phases consisting of water, 10 mM ammonium acetate, methanol, acetonitrile, and isopropanol. The LC system Horizon vanquish was coupled with a Q Exactive Plus Orbitrap mass spectrometer (Thermo Scientific) equipped with a heated electrospray ionization (HESI) source. The resolution was set at 70,000, the scan range was from m/z 150 to 1500, the maximum injection time was 50 ms, the AGC target was 1e6 ions, and the isolation width was 2 Th. Data-dependent analysis (DDA) toN-5 mode was applied using 30% collision energy for higher energy collisional dissociation (HCD). Raw data were analyzed using LipidSearch for peak detection, peak alignment, and lipid identification based on the mass accuracy of the precursor and fragment ions using the lipid blast library database. Differences among cell medium conditions were analyzed based on the relative intensities of the lipids by multiparametric principal component analysis (PCA) and partial least squares discriminant analysis (PLS-DA).

### Cell viability assay (IC50, inhibitory concentration 50)

Cells were seeded (1 × 10^3^) in 96-wells plates (Sarsted, 83.3925) previously treated with Poly-HEMA in the case of suspension cultures. Cells were incubated for 24 h then treated with drug at concentration ranging from 100 μM to 0.001 μM. Dilutions were done in cell culture medium, while highest concentration solution was obtained from drug stock solution in DMSO 10 mM. In the case of lipid particles, they were directly dispersed in cell culture media (CCM) after the purification step, obtaining the highest concentration. Cell viability was assessed after 96 h of exposure time by CellTiter-Glo^®^ Luminescence assay (Promega, WI, USA) for adhesion culture and by Presto Blue^®^ (ThermoFisher, Waltham, Massachusetts) for suspension culture, using plate reader Synergy H1 (Agilent, Santa Clara, California). IC50 values were calculated with Graph Pad Prism 4.0 (Graph Pad Software Corporation, San Diego, CA, USA) from logistical dose–response curves and performed in triplicates^63^.

### Analysis of amiodarone interaction with doxorubicin

Drug combinations effects were evaluated by the simultaneous treatment of A2780 by amiodarone and doxorubicin with concentrations in fixed constant and which corresponds to the same drug efficacy (maximum concentrations were equal to 60uM and 0.053uM respectively for amiodarone and doxorubicin, which then undergoes 1:2 serial dilutions). Cell viability was determined as described in cell viability assay section, drug combination and single drug treatments were performed in the same day in order to compare identical conditions.

Synergistic or antagonistic effect of drug combinations was established by the calculation of combination index (CI) and dose-reduction index (DRI) at increasing fraction affected values (Fa) as described previously^36^, using CompuSyn software (ComboSyn Inc, Paramus, New Jersey).

### Lipidic particles cellular internalisation

DFL, AL and AP were functionalized by the addition of 5.7 µL of rhodamine B 10 mg/ml to 1 ml of AL and AP solutions respectively 1.5 mM of total lipids and 0.65 mM of amiodarone. Solutions were incubated for 24 h at 4 °C, under light exclusion. Purification of functionalized DLF and AL was performed by overnight dialysis, while AP were purified by double centrifugation (3000 *g*, 10 min, 15 °C). A2780 were seeded on a 24 multiwell plate (low density: 15,000 cell/well) and the day after, treated with 0.1 μg/mL of Hoechst for 20 min. Then cells were washed twice with PBS prior to the addition of rhodamine labelled particles. AL and DFL were added at constant rhodamine concentration (3.5 µg/mL), while AP were added at the same concentration of amiodarone of AL (0.6 μM). Indeed, dosing micelle at the same rhodamine concentration of liposomes would determine an excessive content of amiodarone. Images were taken at different time points with Evos^®^ FL Auto Imaging System (ThermoFisher, Waltham, Massachusetts) after cells were washed twice with DPBS and added with 200 µL of DPBS for each well.

### Supplementary Information

Supplementary Information.

## Data Availability

The data supporting the findings of this study are available within the paper, its Supplementary Information files and from the corresponding authors upon reasonable request. Source data are provided with this paper.

## Abbreviations

AFM: Atomic force microscopy
AL: Amiodarone liposomes
ALP: Amiodarone lipidic particles
AP: Amiodarone particles
CPT: Carnitine palmitoyltransferase system
CACT: Carnitine acylcarnitine translocase
DFL: Doxil formulated liposomes
DL: Drug to lipid ratio
DLS: Dynamic light scattering
DPBS/PBS: Dulbecco's phosphate-buffered saline/Phosphate-buffered saline
EE: Encapsulation efficacy
FDA: Food and Drug Administration
FRR: Flow rate ratio
FTIR: Fourier Transform Infrared Spectroscopy
H-NMR: Proton nuclear magnetic resonance
IC50: Half maximal inhibitory concentration
LC: Loading capacity
NPs: Nanoparticles
PCA: Principal component analysis
PdI: Polydispersity index
RT: Room temperature
SD: Standard deviation
TEM: Trasmission electron microscopy
TFR: Total flow rate
