# Supplementary material for: Microfluidic production of amiodarone loaded nanoparticles and application in drug repositioning in ovarian cancer
Source: Sci Rep. 2024 Mar 15;14:6280. doi: 10.1038/s41598-024-55801-3 (PMC10943008; doi:10.1038/s41598-024-55801-3)

# Supplementary information

Peaks assignment………………………………………………………………………………………………….Pag 3

Figure 1S. H-NMR spectra and corresponding molecular structures of lipids used for liposomes production and amiodarone...……………………………………………………………………………………………………....Pag 3

Table 1S. Identified diagnostic peaks used for quantitative H.-NMR…………………………………………….Pag 5

Figure 2S. Temperature effects: DFL produced at different temperature conditions, FFR=3, TFR= 1 ml/min, lipid concentration 10 mM. A) TEM images. B) Size distributions of TEM images measurements; C) DLS analysis: intensity plots of triplicate samples (T1,T2,T3) reported as average of three measurement; D) boxplots of lipids yields; E) Composition analysis: table of lipids percentage composition determined by H-NMR measures (expressed as mol%)………………………………………………………………………………………………………………Pag 6

Table 2S. RSD% of 63_RT triplicate of samples (FFR=3, TFR= 1 ml/min, lipids concentration 10 mM) and derived from the method reproducibility test. RSD% of method reproducibility proved the stability of 63_RT condition…………………………………………………………………………………………………................Pag 7

Figure 3S. FRR effects*:* DFL produced with different FFR, TFR= 1 ml/min, lipids concentration 10 mM, 63_RT *A) TEM images; B)* size distributions of SUV; *C)* DLS analysis: intensity plots of triplicate samples (T1,T2,T3) reported as average of three measurements; D) DLS curve (by volume%) of FRR1 triplicate of samples. TEM image details of FRR1 sample………………………………………………………………………………………………………..Pag 8

Figure 4S. TEM image of DFL sample obtained with 20mM of lipids, FRR2, TFR1 ml/min, 63_RT……………Pag 9

Figure 5S. Z average and PdI data from DLS measurements of triplicates of samples just after their production and over time up to one month; A) DFL samples produced at different FRR, TFR=1 ml/min, lipids concentration 10mM , 63_RT; B) DLF samples produced at different temperature conditions, FRR3, TFR=1ml/min, lipids concentration 10mM. (p-value * <0.05, ** <0.01,***<0.001, ****<0.0001)……………………………………………………………..…Pag 10

Table 3S. The characteristics of Amiodarone predicted in silico (ChemAxon and ALOGPS)………...................Pag 11

Figure 6S. TEM size (above) distributions and average data (below) as effects of different amiodarone concentrations (5 and 10 mM) and FRR (2 and 3) on AL and AP (TFR 1 ml/min, 63_RT). AP distributions are reported for aggregates but also for measure of single NPs. …..……………………………..…………………………………………….Pag 12

Figure 7S. DLS intensity plot A) and respective data B) of a solution of AP (Amiodarone 10mM, TRF=1ml/min, FRR3, total lipids 10mM) at different dilutions………………………………………………………………………..….Pag 13

Table 4S. ALP composition determined by H-NMR measures. Lipid components and amiodarone (DL%) contents are expressed as mol% on total lipids. Reported errors refer to SD…………………………………………………….Pag 14

Table 5S. Lipids and amiodarone yield in ALP determined by H-NMR measures. The reported errors refer to SD………………………………………………………………………………………………………..................Pag 15

Figure 8S. DLS curve (by intensity) of AP samples (A) and AL samples (B) obtained at the three different temperature conditions (63_RT, 63_RT and RT_RT) and with amiodarone concentration equal to 10 and 5 mM (FRR 3, TFR 1 ml/min, lipids 10mM). (T1, T2, T3 are the triplicates of samples reported as average of three measurements) ….Pag 16

Table 6S. Composition of AL determined by H-NMR measures, at three different temperature conditions (63_RT, 63_RT and RT_RT) and with amiodarone concentration equal to 10 and 5 mM (FRR 3, TFR 1 ml/min, lipids 10mM). Lipid components and amiodarone (DL%) contents are expressed as mol% on total lipids. Reported errors refers to SD ……………………………………………………………………………………………………………………...Pag 17

Table 7S. Yields % of AL determined by H-NMR measures, at the three different temperature conditions (63_RT, 63_RT and RT_RT) and with amiodarone concentration equal to 10 and 5 mM (FRR 3, TFR 1 ml/min, lipids 10mM). Reported errors refers to SD ………………………………………………………………………………………Pag 18

Table 8S. Composition % of AP, determined by H-NMR measures, at the three different temperature conditions (63_RT, 63_RT and RT_RT) and with amiodarone concentration equal to 10 and 5 mM (FRR 3, TFR 1 ml/min, lipids 10mM). Lipid components and amiodarone (DL%) contents are expressed as mol% on total lipids. Reported errors refers to SD……………………………………………………………..…………………………………………………...Pag 19

Table 9S. Yields % of AP, determined by H-NMR measures, at the three different temperature conditions (63_RT, 63_RT and RT_RT) and with amiodarone concentration equal to 10 and 5 mM (FRR 3, TFR 1 ml/min, lipids 10mM). Reported errors refers to SD ……………………………………………………………………………………...Pag 20

Figure 9S. Average and PdI data from DLS measurements of triplicates of samples just after their production and over time up to one month. A) AL samples produced at different FRR 3, TFR=1 ml/min, lipids concentration 10mM , 63_RT, amiodarone concentration 5 and 10mM; B) AP samples produced at different temperature conditions, FRR3, TFR=1ml/min, lipids concentration 10mM63_RT, amiodarone concentration 5 and 10mM. (p-value * <0.05, ** <0.01,***<0.001, ****<0.0001; when not reported no significative difference were showed) …………………..Pag 21

Figure 10S. AFM height mode images and cross section profiles of DFL, AL and AP (FRR3, 63_RT, Amiodarone 5mM, TFR 1 ml/min, lipids 10mM)……………..………………………………………………………………………..Pag 22

Figure 11S. Original blots corresponding to crop reported in Figure 4, respectively the first 4 lines of each blot were selected for the image. Biological triplicates were run for each considered condition and were processed in parallel (Oa OVACR-5 adhesion; Os OVACR-5 suspension; Ka Kuramochi adhesion; Ks Kuramochi suspension; Aa A2780 adhesion; As A2780 suspension). A) Blot corresponding to 25ug of protein loading B) Blot corresponding to 40ug of protein loading. Additional uncharacterized band around 60kDa was also observed across the samples tested, which was particularly clear for higher protein loading…………………………………………………………………………Pag23

Figure 12S. PCA score plots (top) and dendrograms of hierarchical clustering (bottom) obtained from FT-IR spectra of cells grown in adhesion (adh) and suspension (susp) condition. Hierarchical clustering of A2780 and Kuramochi using Pearson’s correlation for similarity measures, while OVCAR-5 applied Euclidean distance. Ward's linkage was used as clustering algorithm for all the cell lines…………………………………………………………………………...Pag 24

Figure 13S. PCA of lipidomics profile of amiodarone treated A2780, acquired in positive and negative mode respectively on left and right side…………………………………………..……………………………………….Pag 25

Table 10S. IC50 values of amiodarone and cisplatin for adhesion (adh) and suspension (susp) cultures of Kuramochi, A2780, OVCAR-5. Reported errors refers to standard deviation (SD)……………………………………………..Pag26

Table 11S. Comparison of IC50 values of free drug and NPs formulations. Reported errors refers to SD………………………………………………………………………………………………………………..…Pag27

Figure 14S. Evaluation of amiodarone combi9nation with doxorubicin. Logarithmic Fa-CI and Fa-DRI plots obtained by CompuSyn software……………………………………………………………………………………………..Pag28

Peaks assignment

H-NMR spectra of pure compound were measured in order to assign peaks and select diagnostic ones to quantify samples’ components.


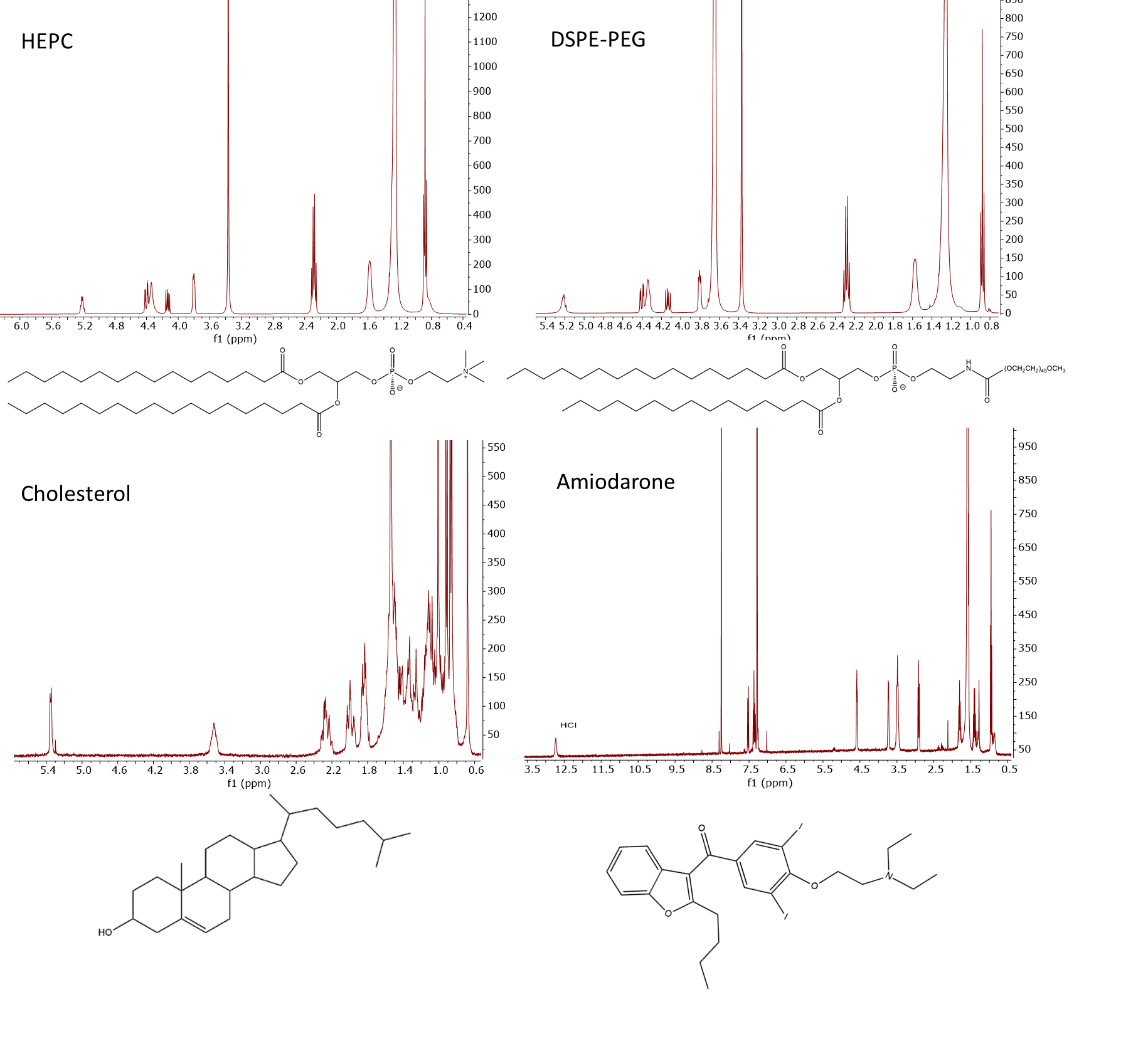


**Figure 1S**. H-NMR spectra and corresponding molecular structures of lipids used for liposomes production and amiodarone

HEPC: ^1^H NMR (400 MHz, CDCl_3_) δ 5.22 (m, *J* = 5.7, 3.1 Hz, 1H), 4.40 (dd, *J* = 12.0, 3.0 Hz, 2H), 4.34 (s, 2H), 4.13 (dd, *J* = 12.0, 7.2 Hz, 2H), 3.80 (t, *J* = 4.8 Hz, 2H), 3.37 (s, 9H), 2.28 (q, *J* = 7.8 Hz, 4H), 1.58 (s, 4H), 1.26 (d, *J* = 6.4 Hz, 52H), 0.93 – 0.84 (m, 6H).

DSPE-PEG: 1H NMR (400 MHz, ) δ 5.21 (dq, J = 8.0, 5.2 Hz, 1H), 4.40 (dd, J = 12.0, 3.0 Hz, 2H), 4.33 (s, J = 8.5 Hz, 2H), 4.13 (dd, J = 12.1, 7.2 Hz, 2H), 3.81 (dd, J = 6.0, 3.4 Hz, 1H), : 3.63 (s, 180H) 3.37 (s, 9H), 2.28 (q, J = 7.7 Hz, 4H), 1.70 – 1.49 (m, 4H), 1.25 (s, 40H), 0.94 – 0.83 (m, 6H).

CHO: 1H NMR (400 MHz, CDCl3) δ 5.48 – 5.23 (m, 1H), 3.52 (s, 1H), 2.39 – 2.12 (m, 2H), 2.06 – 1.78 (m, 4H), 1.68 – 0.93 (m, 26), 0.92 (d, J = 6.4 Hz, 3H), 0.87 (m, J = 6.6, 1.8 Hz, 4H), 0.68 (s, 3H).

Amiodarone: 1H NMR (400 MHz, CDCl3) δ 8.25 (s, 2H), 7.56 – 7.49 (m, 1H), 7.36 (q, J = 8.0 Hz, 2H), 4.58 (t, J = 4.8 Hz, 2H), 3.73 (d, J = 5.0 Hz, 2H), 3.56 – 3.43 (m, 4H), 2.96 – 2.87 (m, 2H), 1.81 (p, J = 7.6 Hz, 2H), 1.58 (d, J = 6.0 Hz, 9H), 1.40 (h, J = 7.4 Hz, 2H), 1.29 (d, J = 11.3 Hz, 2H), 0.95 (t, J = 7.3 Hz, 3H).

Selected diagnostic peaks for all the components of amiodarone loaded liposome are reported in Table 1S (HEPC quantification was corrected considering the contribution of DSPE-PEG).

**Table 1S.** *Identified diagnostic peaks used for quantitative H-NMR.*

| **Compound** | **δ (ppm)** | **n° H** | **multiplicity** |
| --- | --- | --- | --- |
| Cholesterol | 0.68 | 3 | singlet |
| DSPE-PEG | 3.63 | 180 | multiplet |
| HEPC | 5.22 | 1 | multiplet |
| Amiodarone | 8.25 | 2 | singlet |

*
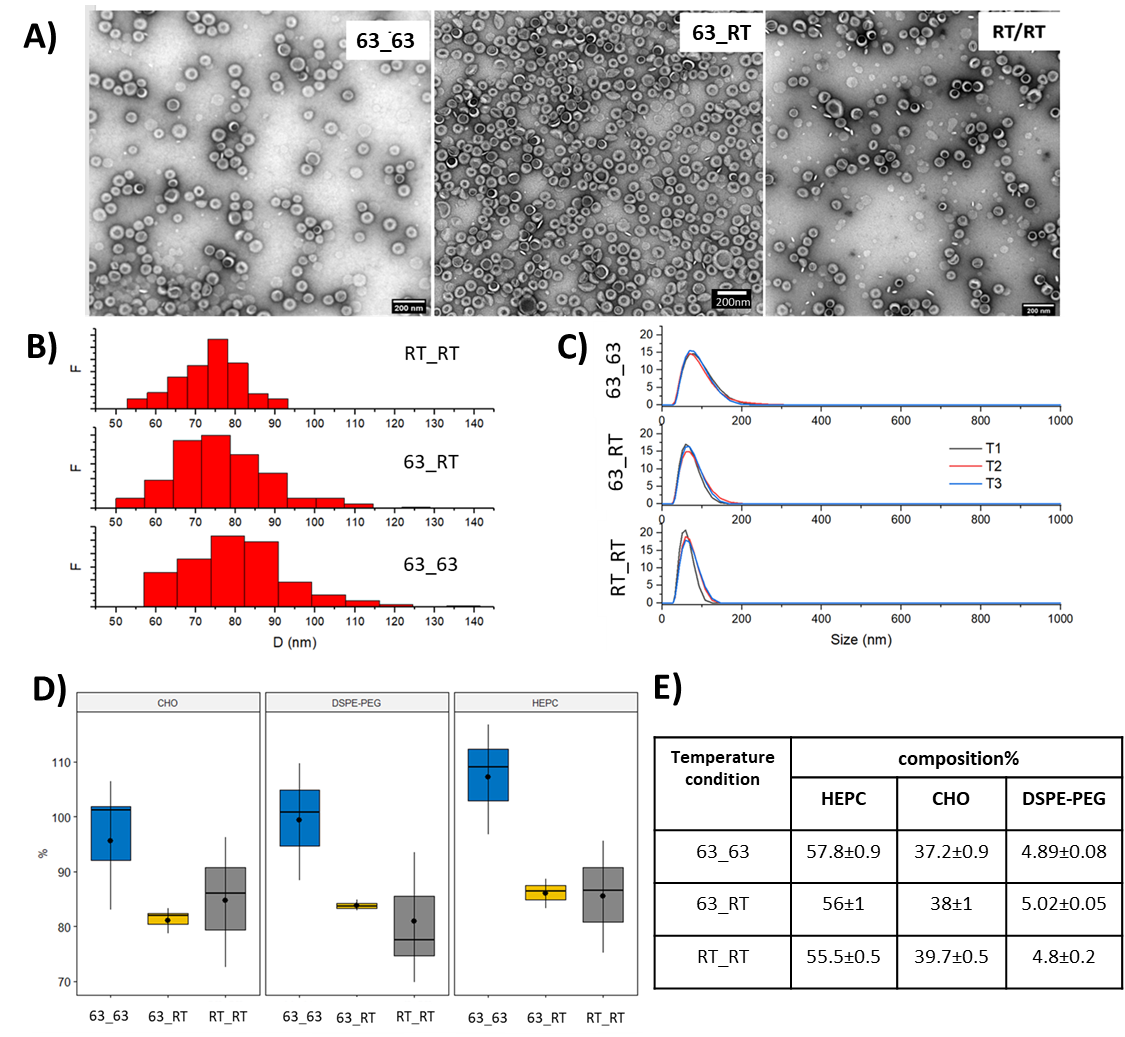
*

***Figure 2S****. Temperature effects:* *DFL produced at different temperature conditions, FFR3:1, TFR= 1 ml/min, lipid concentration 10 mM. A) TEM images. B) Size distributions of TEM images measurements; C) DLS analysis: intensity plots* *of triplicate samples (T1,T2,T3) reported as average of three measurement; D) boxplots of lipids yields; E) Composition analysis: table of lipids percentage composition* *determined by H-NMR measures (expressed as mol%).*

***Table 2S****. Relative standard deviation percentage (RSD%) of 63_RT triplicate of samples (FFR=3:1, TFR= 1 ml/min, lipids concentration 10 mM) and derived from the method reproducibility test. RSD% of method reproducibility proved the stability of 63_RT condition.*

| **RSD%** | **HEPC** | **DSPE-PEG** | **CHO** |
| --- | --- | --- | --- |
| **63_RT samples** | 1.1 | 2.9 | 2.6 |
| **Method reproducibility** | 3.3 | 1.9 | 5.7 |


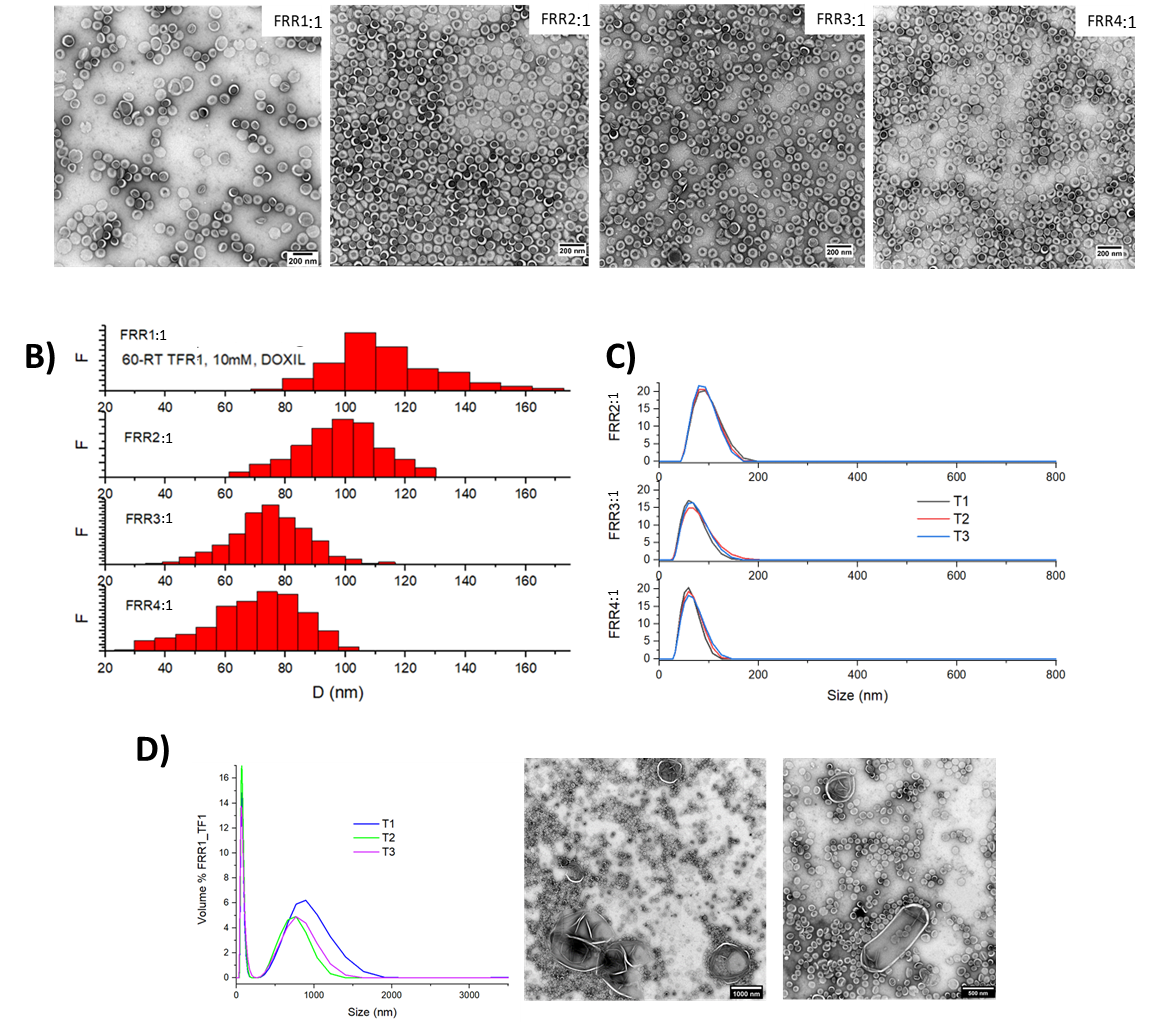


**Figure 3S.** FRR effects: DFL produced with different FFR, TFR= 1 ml/min, lipids concentration 10 mM, 63_RT A) TEM images; B) size distributions of SUV; C) DLS analysis: intensity plots of triplicate samples (T1,T2,T3) reported as average of three measurements; D) DLS curve (by volume%) of FRR1 triplicate of samples. TEM image details of FRR1:1 sample.


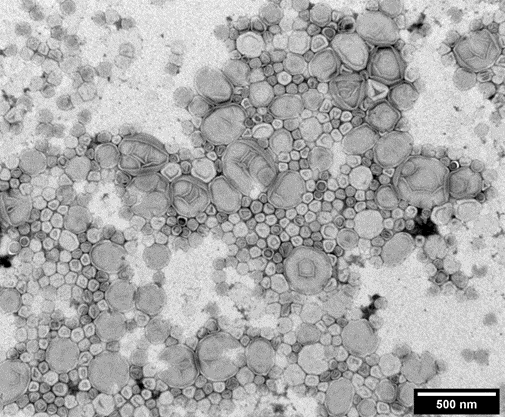


***Figure 4S.*** *TEM image of DFL sample obtained with 20Mm of lipids, FRR2:1, TFR1 ml/min, 63_RT.*


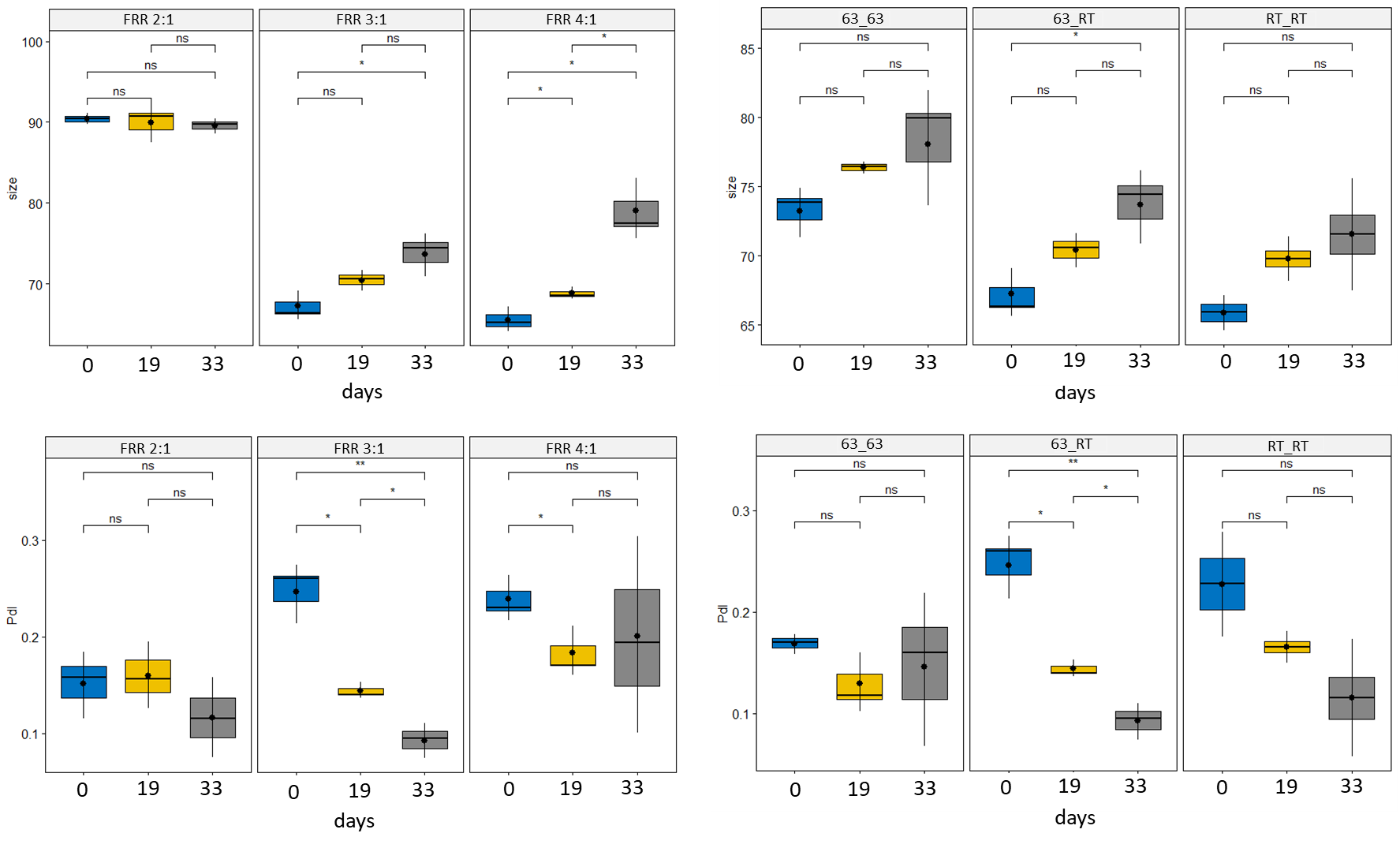
***Figure 5S.*** *Z average and PdI data from DLS measurements of triplicates of samples just after their production and over time up to one month; A) DFL samples produced at different FRR, TFR=1 ml/min, lipids concentration 10mM , 63_RT; B) DLF samples produced at different temperature conditions, FRR3:1, TFR=1ml/min, lipids concentration 10mM. (p-value * <0.05, ** <0.01,***<0.001, ****<0.0001).*

**Table 3S.** The characteristics of Amiodarone predicted in silico (ChemAxon and ALOGPS).

| Molecular weight (neutral) | 645.3 |
| --- | --- |
| pKa (strongest basic, (protonated) ammine) | 8.47 ([ChemAxon](https://chemaxon.com/)) |
| Log P | 7.24 (ALOGPS) |
| Log S | -5.1 (ALOGPS) |
| Water solubility | 0.00476 mg/ml (ALOGPS) |


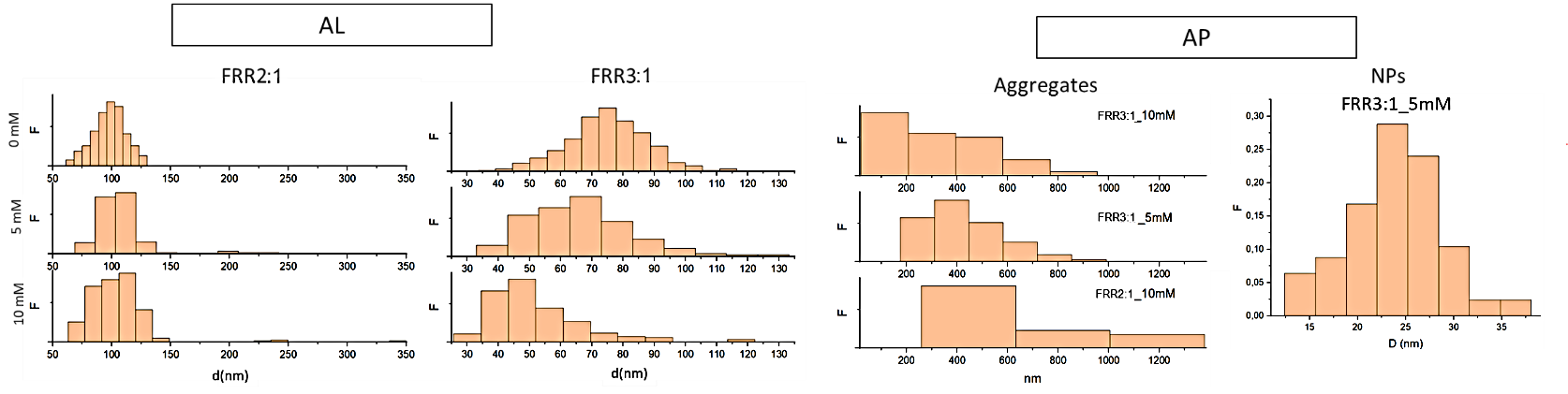


| **NPs** | **FRR** | **AM (mM)** | **Size (nm)** |
| --- | --- | --- | --- |
| AL | 3:1 | 5 | 67±1 |
| AL | 3:1 | 10 | 52±1 |
| AL | 2:1 | 5 | 112±2 |
| AL | 2:1 | 10 | 112±3 |
| AP (aggregate) | 3:1 | 5 | 290±30 |
| AP (aggregate) | 3:1 | 10 | 435±19 |
| AP (aggregate) | 2:1 | 10 | 604±62 |
| AP (particles) | 3:1 | 5 | 24±5 |

**Figure 6S.** TEM size (above) distributions and average data (below) as effects of different amiodarone concentrations (5 and 10 mM) and FRR (2:1 and 3:1) on AL and AP (TFR 1 ml/min, 63_RT). AP distributions are reported for aggregates but also for measure of single NPs.


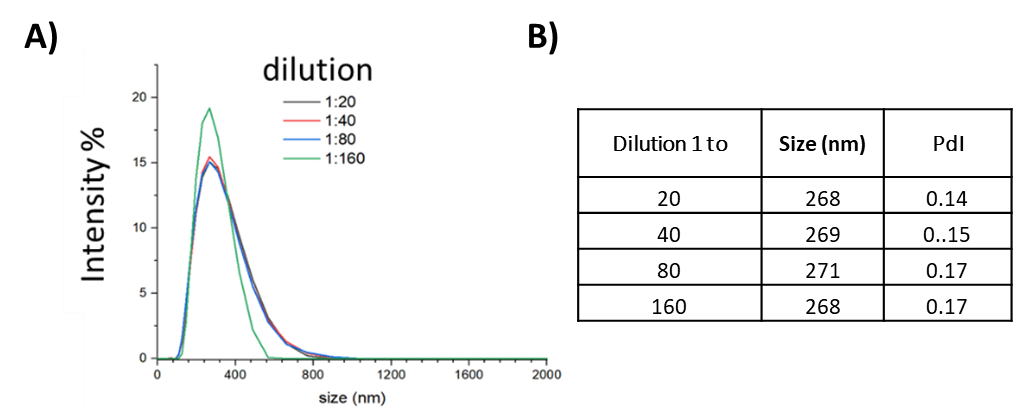


***Figure 7S.*** *DLS intensity plot A) and respective data B) of a solution of amiodarone particles AP (Amiodarone 10mM, TRF=1ml/min, FRR3:1, total lipids 10mM) at different dilutions.*

**Table 4S.** Amiodarone lipidic particles (ALP) (liposomes (AL) and particles (AP)) composition determined by H-NMR measures. Lipid components and amiodarone (DL%) contents are expressed as mol% on total lipids. Reported errors refer to SD.

| **Production parameters** | | **Sample fraction** | **Obtained particles composition** | | | |
| --- | --- | --- | --- | --- | --- | --- |
| **Amiodarone(mM)** | **FRR** | **NPs** | **HEPC** | **DSPE-PEG** | **CHO** | **DL %** |
| 5 | 3:1 | AL | 54±4 | 5.0±0.5 | 37±4 | 16±4 |
| 10 | 3:1 | AL | 54±1 | 5.6±0.2 | 40±1 | 16±3 |
| 5 | 2:1 | AL | 53±4 | 6±1 | 41±3 | 1±0.3 |
| 10 | 2:1 | AL | 53.6±0.5 | 5.6±0.2 | 41±1 | 2± 1 |
| 5 | 3:1 | AP | - | 3±1 | 97±1 | 1.6±0.5x 10^3^ |
| 10 | 3:1 | AP | - | 2.8±0.8 | 97.2±0.8 | 1.93±0.07x10^3^ |
| 5 | 2:1 | AP | - | 0.8±0.1 | 99.2±0.1 | 1.4±0.2 x10^3^ |
| 10 | 2:1 | AP | - | 1.0±0.1 | 99.0±0.8 | 2.0±0.1 x10^3^ |

***Table 5S.*** *Lipids and amiodarone yield in ALP determined by H-NMR measures. The reported errors refer to SD.*

| **Production parameters** | | **Sample fraction** | **Yields** | | | | |
| --- | --- | --- | --- | --- | --- | --- | --- |
| **Amiodarone(mM)** | **FRR** | **NPs** | **CHO** | **DSPE-PEG** | **HEPC** | **Total lipids** | **Amiodarone (EE%)** |
| 5 | 3:1 | AL | 60 ± 10 | 60 ± 20 | 60 ± 20 | 60 ± 10 | 21 ± 4 |
| 10 | 3:1 | AL | 50 ± 10 | 60 ± 10 | 60 ± 10 | 50 ± 10 | 18 ± 8 |
| 5 | 2:1 | AL | 60 ± 10 | 70 ± 20 | 65 ± 2 | 60 ± 5 | 1.0 ± 0.4 |
| 10 | 2:1 | AL | 63 ± 9 | 70 ± 10 | 70 ± 10 | 62 ± 9 | 1.2 ± 0.8 |
| 5 | 3:1 | AP | 5 ± 5 | 0.8±0.1 | - | 1.3 ± 0.1 | 38 ± 5 |
| 10 | 3:1 | AP | 8.2 ± 0.8 | 1.9±0.4 | - | 3.4 ± 0.3 | 65 ± 3 |
| 5 | 2:1 | AP | 6 ± 1 | 0.4±0.2 | - | 2.6 ± 0.6 | 73 ± 26 |
| 10 | 2:1 | AP | 10 ± 3 | 0.9±0.3 | - | 4 ± 1 | 81 ± 20 |


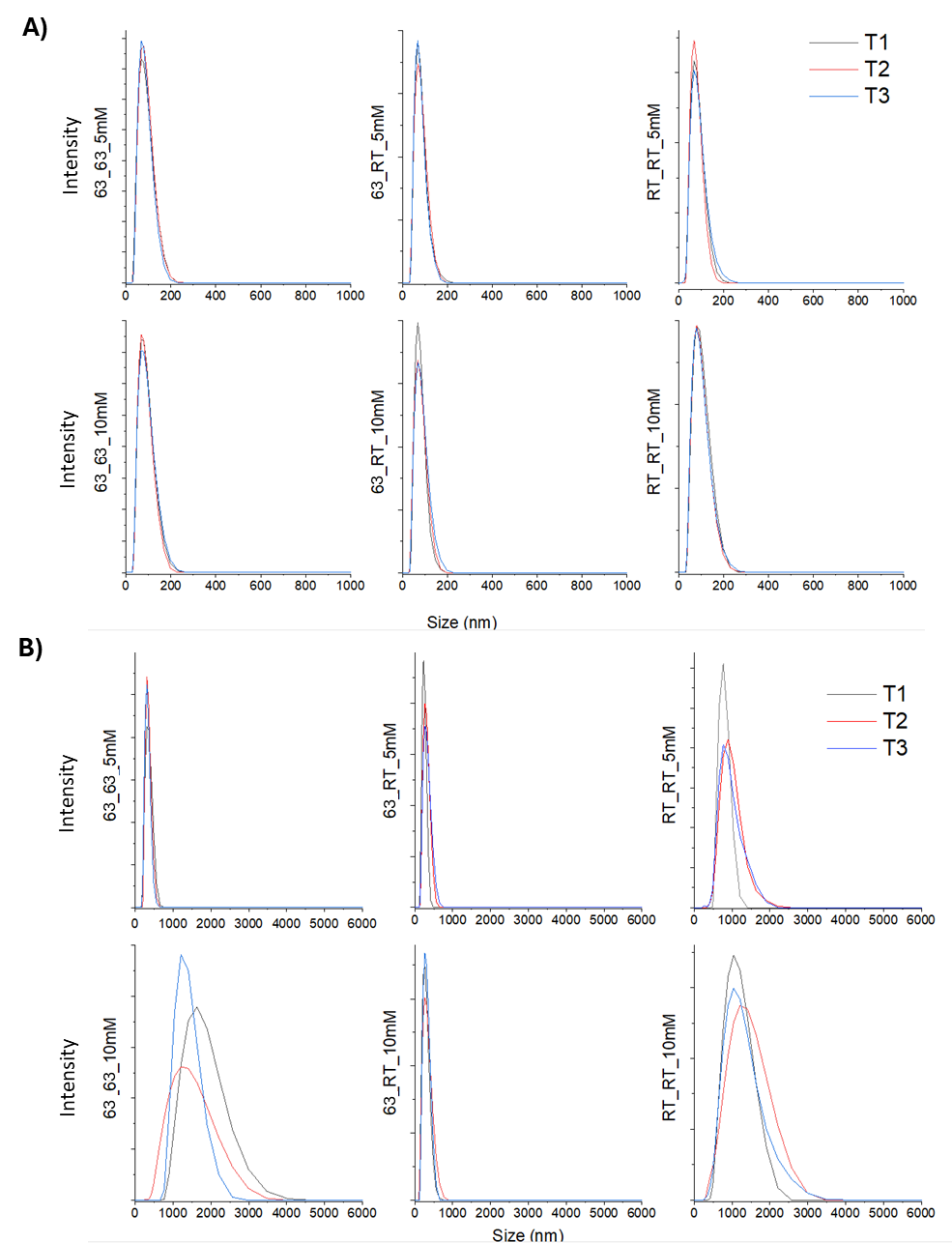


**Figure** **8S.** DLS curve (by intensity) of amiodarone liposome (AL) samples (A) and amiodarone particles (AP) samples (B) obtained at the three different temperature conditions (63_RT, 63_RT and RT_RT) and with amiodarone production concentration equal to 10 and 5 mM (FRR 3:1, TFR 1 ml/min, lipids 10mM). (T1, T2, T3 are the triplicates of samples reported as average of three measurements).

***Table 6S.*** *Composition of AL determined by H-NMR measures, at three different temperature conditions (63_RT, 63_RT and RT_RT) and with amiodarone concentration equal to 10 and 5 mM (FRR 3:1, TFR 1 ml/min, lipids 10mM). Lipid components and amiodarone (DL%) contents are expressed as mol% on total lipids. Reported errors refers to SD.*

| **Production parameters** | | **Obtained liposomes composition** | | | |
| --- | --- | --- | --- | --- | --- |
| **Amiodarone(mM)** | **Temperature** | **CHO** | **DSPE-PEG** | **HEPC** | **DL (%)** |
| 5 | 63_63 | 34±2 | 5.0±0.3 | 61±2 | 10±1 |
| 10 | 63_63 | 34±3 | 5.0±0.5 | 61±3 | 8±1 |
| 5 | 63_RT | 40±2 | 5.4±0.1 | 54±2 | 18±4 |
| 10 | 63_RT | 40±1 | 5.6±0.2 | 54±1 | 16±3 |
| 5 | RT_RT | 38±1 | 4.9±0.2 | 58±2 | 17±3 |
| 10 | RT_RT | 35±3 | 4.8±0.1 | 60±3 | 11±1 |

***Table 7S.*** *Yields % of AL determined by H-NMR measures, at the three different temperature conditions (63_RT, 63_RT and RT_RT) and with amiodarone concentration equal to 10 and 5 mM (FRR 3:1, TFR 1 ml/min, lipids 10mM). Reported errors refers to SD.*

| **Production parameters** | | **Yields** | | | | |
| --- | --- | --- | --- | --- | --- | --- |
| **Amiodarone (mM)** | **Temperature** | **CHO** | **DSPE-PEG** | **HEPC** | **Total lipids** | **Amiodarone (EE%)** |
| 5 | 63_63 | 45±13 | 53±12 | 55±16 | 51±14 | 10±1 |
| 10 | 63_63 | 34±11 | 40±14 | 43±10 | 39±10 | 3±1 |
| 5 | 63_RT | 62±16 | 66±18 | 66±18 | 61±16 | 21±5 |
| 10 | 63_RT | 55±14 | 61±12 | 59±13 | 55±13 | 18±8 |
| 5 | RT_RT | 45±4 | 48±3 | 52±3 | 48±2 | 16±4 |
| 10 | RT_RT | 47±8 | 53±6 | 59±3 | 54±5 | 6±1 |

***Table 8S.*** *Composition % of AP, determined by H-NMR measures, at the three different temperature conditions (63_RT, 63_RT and RT_RT) and with amiodarone concentration equal to 10 and 5 mM (FRR 3:1, TFR 1 ml/min, lipids 10mM). Lipid components and amiodarone (DL%) contents are expressed as mol% on total lipids. Reported errors refers to SD.*

| **Production parameters** | | **Obtained particles composition** | | |
| --- | --- | --- | --- | --- |
| **Amiodarone(mM)** | **Temperature** | **CHO** | **DSPE-PEG** | **DL (%)** |
| 5 | 63_63 | 97.3±0.2 | 2.7±0.2 | 1930±159 |
| 10 | 63_63 | 97.0± 0.5 | 3.0±0.5 | 2477 ± 323 |
| 5 | 63_RT | 97±1 | 3±1 | 1743±362 |
| 10 | 63_RT | 97.2±0.8 | 2.8±0.8 | 1930 ± 67 |
| 5 | RT_RT | 96.1±0.7 | 96.1±0.7 | 1569 ±279 |
| 10 | RT_RT | 96.5±0.4 | 96.5±0.4 | 2180 ±104 |

***Table 9S.*** *Yields % of AP, determined by H-NMR measures, at the three different temperature conditions (63_RT, 63_RT and RT_RT) and with amiodarone concentration equal to 10 and 5 mM (FRR 3:1, TFR 1 ml/min, lipids 10mM). Reported errors refers to SD.*

| **Production parameters** | | **Obtained particles composition** | | | |
| --- | --- | --- | --- | --- | --- |
| **Amiodarone (mM)** | **Temperature** | **CHO** | **DSPE-PEG** | **Total lipids** | **Amiodarone (EE%)** |
| 5 | 63_63 | 3±0.4 | 0.65±0.08 | 1.2±0.2 | 48±10 |
| 10 | 63_63 | 4.9±0.2 | 1.20±0.22 | 2.00±0.09 | 49±6 |
| 5 | 63_RT | 3.0±0.3 | 0.8±0.1 | 1.3±0.1 | 38±5 |
| 10 | 63_RT | 8.2±0.8 | 1.9±0.4 | 3.4±0.3 | 65±3 |
| 5 | RT_RT | 2.0±0.2 | 0.74±0.06 | 0.8±0.1 | 24±4 |
| 10 | RT_RT | 6.1±0.1 | 1.8±0.2 | 2.55±0.04 | 57±3 |


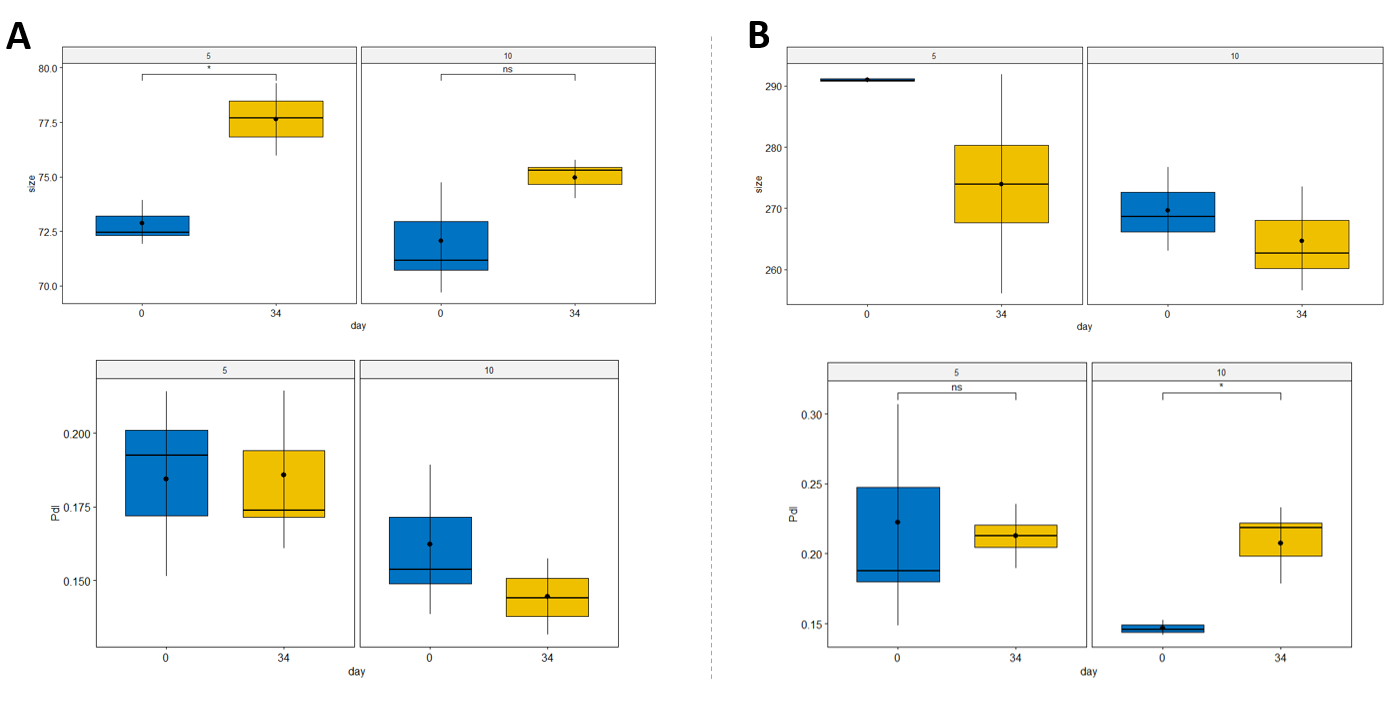


**Figure 9S.** Average and PdI data from DLS measurements of triplicates of samples just after their production and over time up to one month. A) amiodarone liposome (AL) samples produced at different FRR 3, TFR=1 ml/min, lipids concentration 10mM , 63_RT, amiodarone concentration 5 and 10mM; B) amiodarone particle (AP) samples produced at different temperature conditions, FRR3:1, TFR=1ml/min, lipids concentration 10mM63_RT, amiodarone concentration 5 and 10mM. (p-value * <0.05, ** <0.01,***<0.001, ****<0.0001; when not reported no significative difference were showed).


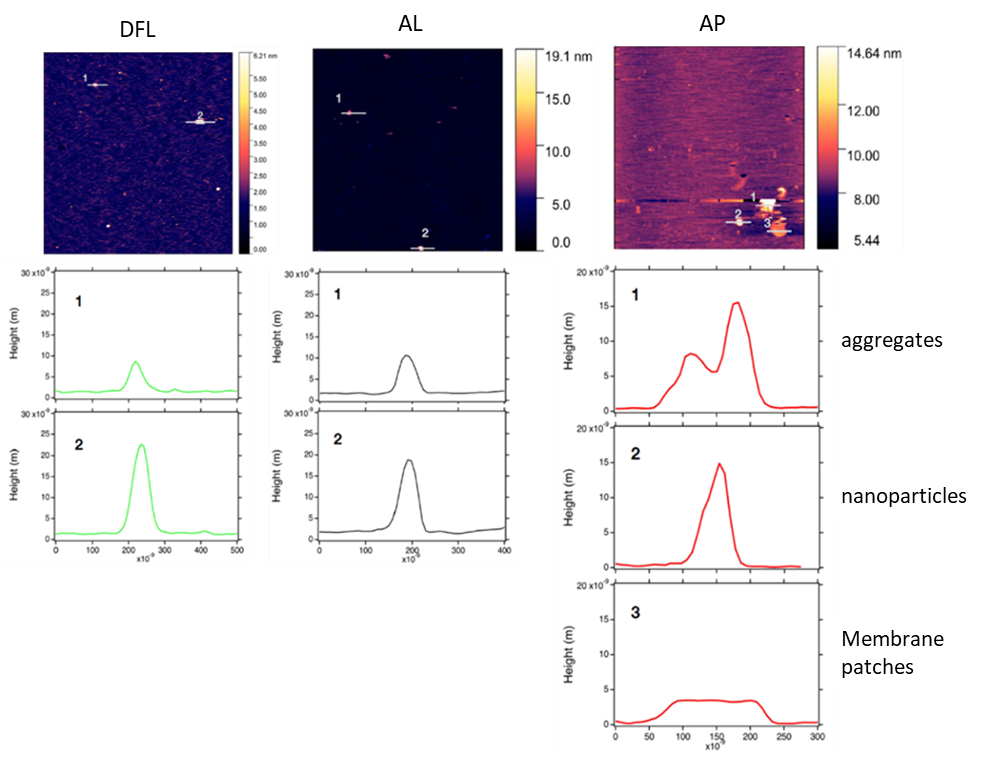


**Figure 10S.** AFM height mode images and cross section profiles of Doxil formulated liposomes (DFL), amiodarone liposomes (AL) and amiodarone particles (AP) (FRR3:1, 63_RT, Amiodarone 5mM, TFR 1 ml/min, lipids 10mM).


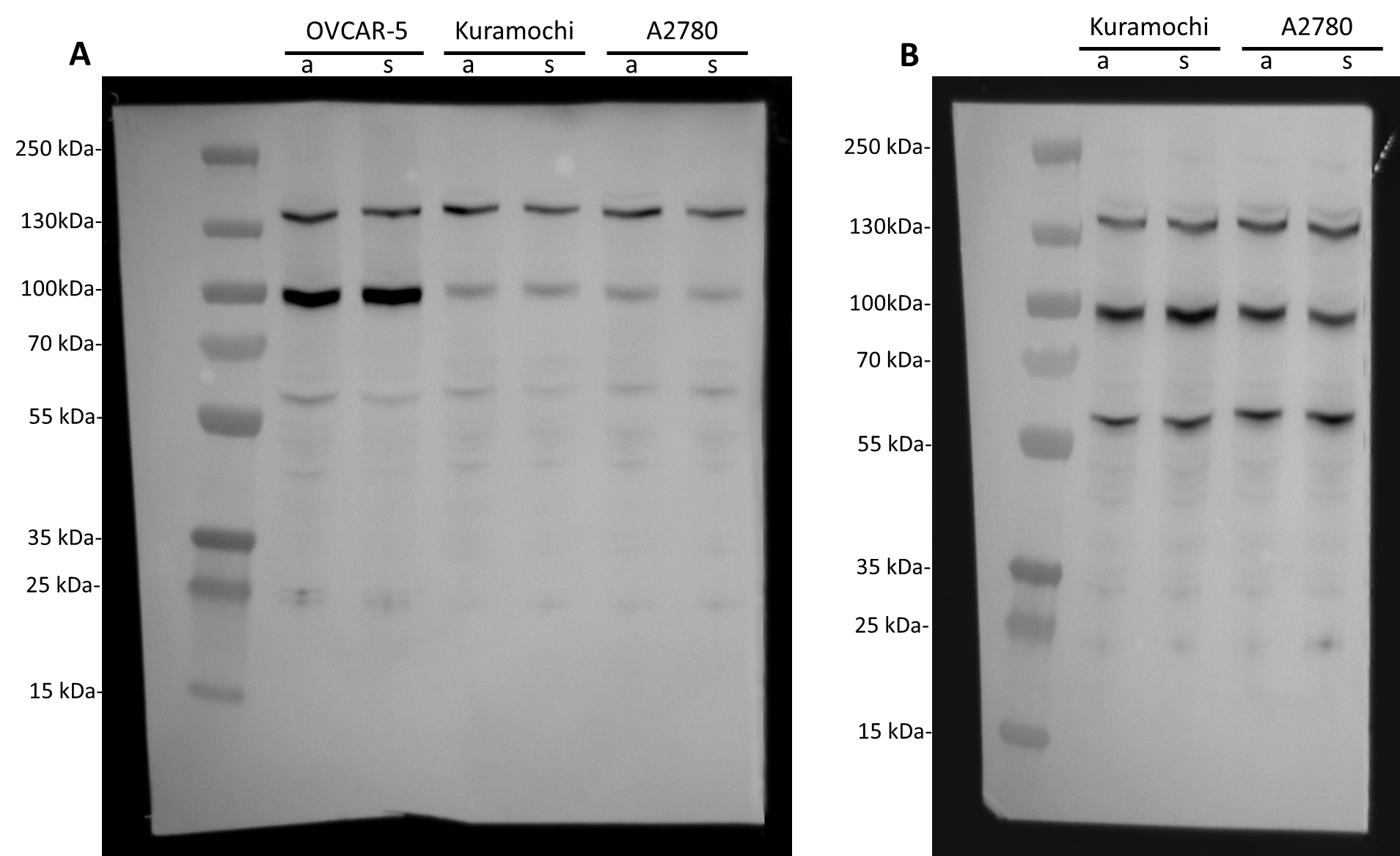


**Figure 11S** Original blots corresponding to crop reported in Figure 4. Label “a” is related to adhesion cultures while label “s” refers to suspension cultures. (A) Blot corresponding to 25ug of protein loading B) Blot corresponding to 40ug of protein loading. Additional uncharacterized band around 60kDa was also observed across the samples tested, which was particularly clear for higher protein loading.


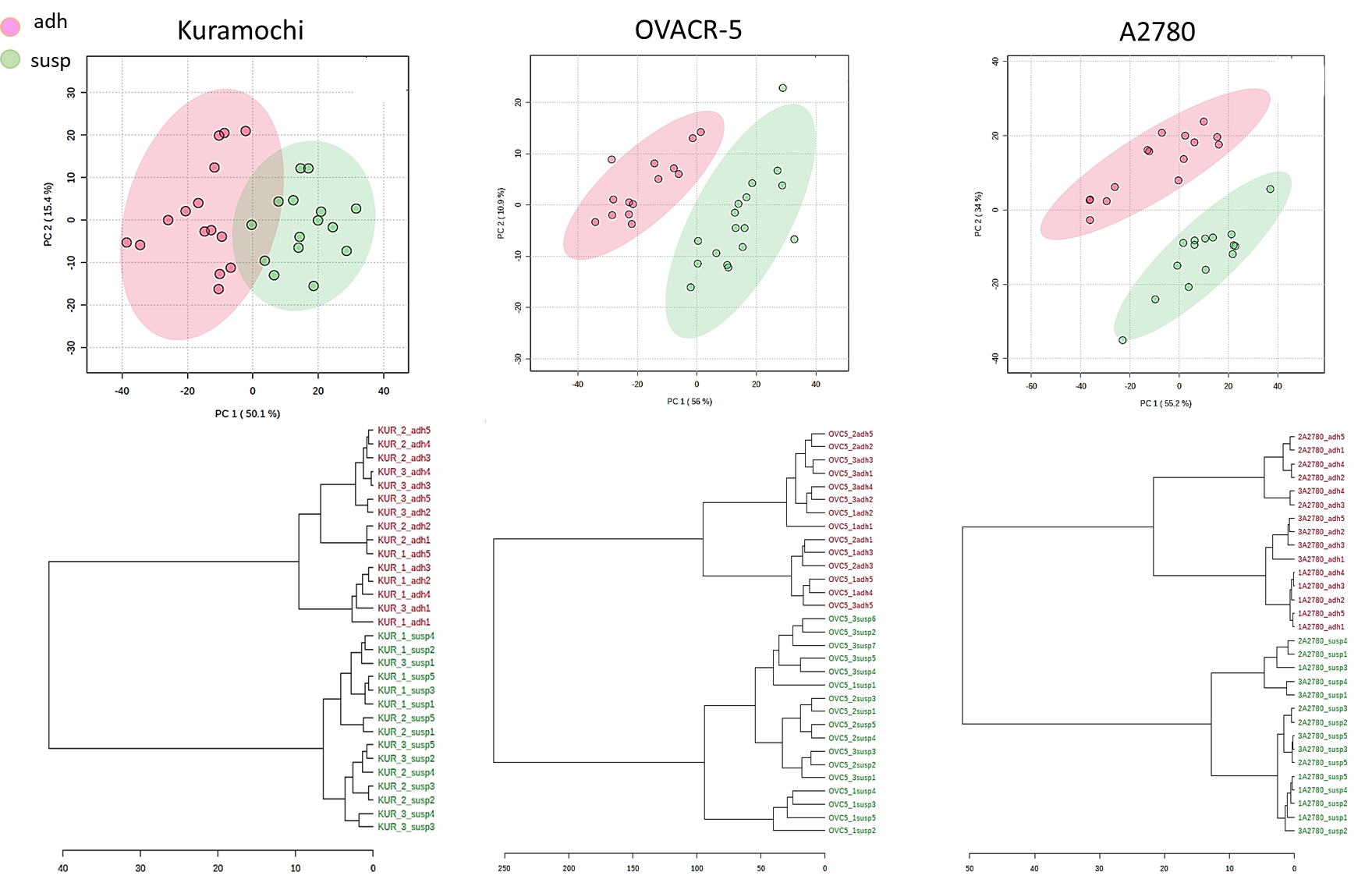


**Figure 12S.** PCA score plots (top) and dendrograms of hierarchical clustering (bottom) obtained from FT-IR spectra of cells grown in adhesion (adh) and suspension (susp) condition. Hierarchical clustering of A2780 and Kuramochi using Pearson’s correlation for similarity measures, while OVCAR-5 applied Euclidean distance. Ward's linkage was used as clustering algorithm for all the cell lines.


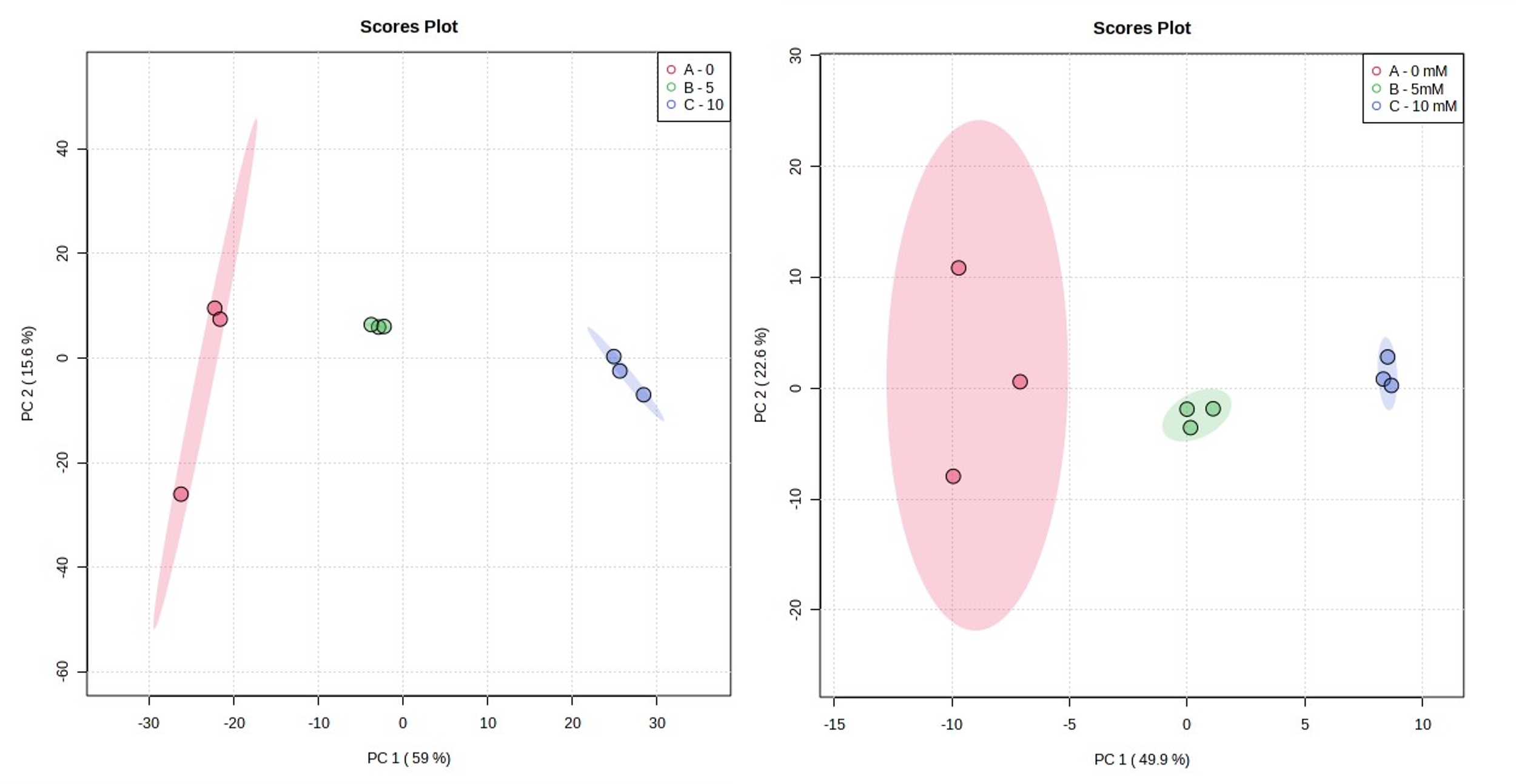


**Figure 13S.** PCA of lipidomics profile of amiodarone treated A2780, acquired in positive and negative mode respectively on left and right side.

**Table 10S.** IC50 values of amiodarone and cisplatin for adhesion (adh) and suspension (susp) cultures of Kuramochi, A2780, OVCAR-5. Reported errors refers to standard deviation (SD)

| **IC50 (μM)** | **Amiodarone** | | **Cisplatin** | |
| --- | --- | --- | --- | --- |
| **Cell line** | **Adh** | **Susp** | **Adh** | **Susp** |
| Kuramochi | 5.6 ± 0.3 | 25 ± 7 | 1.1±0.2 | 0.9±0.3 |
| OVCAR-5 | 3.2 ± 0.4 | 6.7 ± 0.8 | 1.0±0.2 | 0.7±0.2 |
| A2780 | 1.5 ± 0.5 | 4.7 ± 1 | 0.44±0.02 | 0.51±0.01 |

**Table 11S.** Comparison of IC50 values of free drug and lipidic nanoparticles formulations. Reported errors refers to SD.

|  |  | **IC50 (µM)** | | |
| --- | --- | --- | --- | --- |
| **treatment** | **culture** | **Kuramochi** | **OVCAR-5** | **A2780** |
| drug | adh | 5.6 ±0.3 | 3.2±0.5 | 1.5±0.5 |
| AP | adh | 3±1 | 0.4±0.1 | 2.0±0.5 |
| AL | adh | 1.3±0.4 | 1.4±0.4 | 1.5±0.5 |
| drug | susp | 25±7 | 6.7±0.83 | 5±1 |
| AP | sup | 6±1 | 2±1 | 3±1 |
| AL | sup | >100 | 41±3 | 30±5 |

**Figure 14S.** Evaluation of amiodarone combi9nation with doxorubicin. Logarithmic Fa-CI and Fa-DRI plots obtained by CompuSyn software.


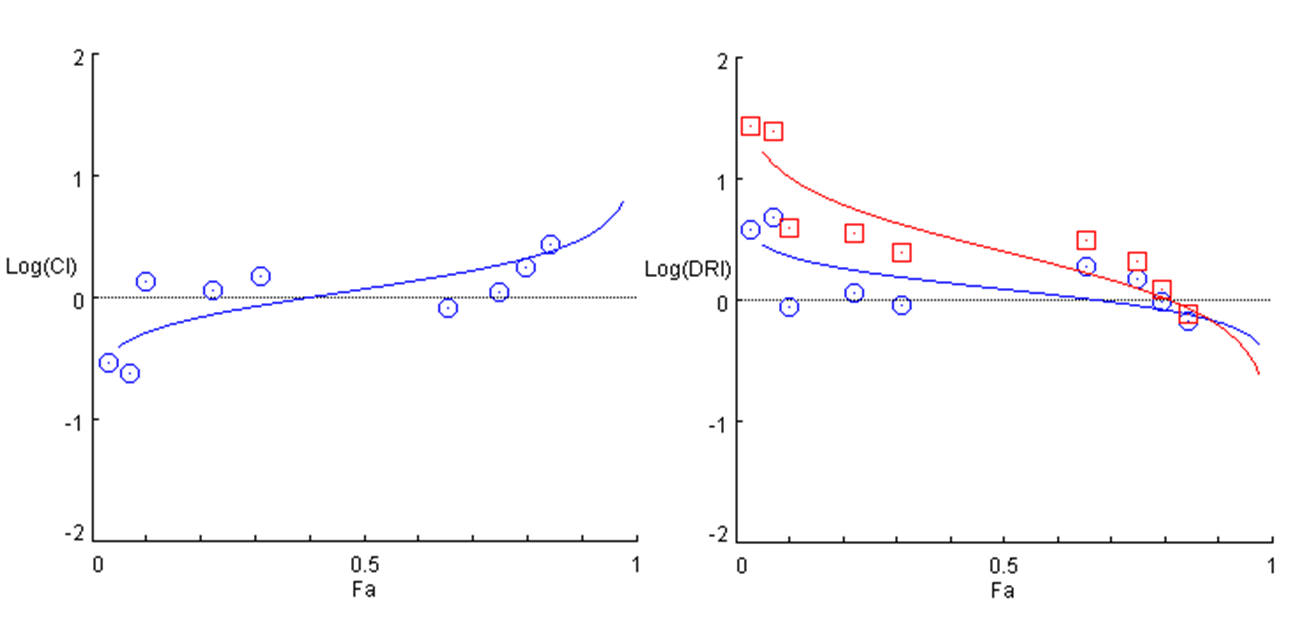

Supplement: Supplementary file 1 — Supplementary Information. [file 41598_2024_55801_MOESM1_ESM.docx]
